# Tandem metabolic reaction–based sensors unlock in vivo metabolomics

**DOI:** 10.1073/pnas.2425526122

**Published:** 2025-02-27

**Authors:** Xuanbing Cheng, Zongqi Li, Jialun Zhu, Jingyu Wang, Ruyi Huang, Lewis W. Yu, Shuyu Lin, Sarah Forman, Evelina Gromilina, Sameera Puri, Pritesh Patel, Mohammadreza Bahramian, Jiawei Tan, Hannaneh Hojaiji, David Jelinek, Laurent Voisin, Kristie B. Yu, Ao Zhang, Connie Ho, Lei Lei, Hilary A. Coller, Elaine Y. Hsiao, Beck L. Reyes, Joyce H. Matsumoto, Daniel C. Lu, Chong Liu, Carlos Milla, Ronald W. Davis, Sam Emaminejad

**Affiliations:** ^a^Interconnected and Integrated Bioelectronics Lab (I²BL), Department of Electrical and Computer Engineering, Samueli School of Engineering, University of California, Los Angeles, CA 90095; ^b^Department of Materials Science and Engineering, Samueli School of Engineering, University of California, Los Angeles, CA 90095; ^c^Department of Chemistry and Biochemistry, University of California, Los Angeles, CA 90095; ^d^Department of Neurosurgery, David Geffen School of Medicine, University of California, Los Angeles, CA 90095; ^e^Neuromotor Recovery and Rehabilitation Center, David Geffen School of Medicine, University of California, Los Angeles, CA 90095; ^f^Brain Research Institute, University of California, Los Angeles, CA 90095; ^g^Department of Integrative Biology and Physiology, University of California, Los Angeles, CA 90095; ^h^Department of Molecular, Cell and Developmental Biology, University of California, Los Angeles, CA 90095; ^i^Interdepartmental Program in Neuroscience, University of California, Los Angeles, CA 90095; ^j^Institute for Society and Genetics, University of California, Los Angeles, CA 90095; ^k^Department of Mechanical and Aerospace Engineering, University of California, Los Angeles, CA 90095; ^l^Department of Biological Chemistry, David Geffen School of Medicine, University of California, Los Angeles, CA 90095; ^m^Molecular Biology Institute, University of California, Los Angeles, CA 90095; ^n^Division of Pediatric Neurology, Department of Pediatrics, University of California, Los Angeles, CA 90095; ^o^The Stanford Cystic Fibrosis Center, Center for Excellence in Pulmonary Biology, Stanford University School of Medicine, Stanford, CA 94305; ^p^Stanford Genome Technology Center, Stanford University School of Medicine, Stanford, CA 94304; ^q^Department of Bioengineering, University of California, Los Angeles, CA 90095

**Keywords:** wearable and implantable metabolite sensors, cofactor-assisted enzymatic reactions, in vivo metabolomics, microbiome, personalized medicine

## Abstract

Mimicking metabolic pathways on electrodes enables in vivo metabolite monitoring for decoding metabolism. Conventional in vivo sensors cannot accommodate underlying complex reactions involving multiple enzymes and cofactors, addressing only a fraction of enzymatic reactions for few metabolites. We devised a single-wall-carbon-nanotube-electrode architecture supporting tandem metabolic pathway–like reactions linkable to oxidoreductase-based electrochemical analysis, making a vast majority of metabolites detectable in vivo. This architecture robustly integrates cofactors, self-mediates reactions at maximum enzyme capacity, and facilitates metabolite intermediation/detection and interference inactivation through multifunctional enzymatic use. Accordingly, we developed sensors targeting 12 metabolites, with 100-fold-enhanced signal-to-noise ratio and days-long stability. Leveraging these sensors, we monitored trace endogenous metabolites in sweat/saliva for noninvasive health monitoring, and a bacterial metabolite in the brain, marking a key milestone for unraveling gut microbiota–brain axis dynamics.

## Significance

This work presents a sensor design that harnesses naturally proven metabolic pathways and evolutionarily robust molecular toolkits (enzymes and cofactors) for reliable, real-time, and continuous in vivo monitoring of a vast majority of metabolites. The architecture is based on a multifunctional single-wall-carbon-nanotube electrode that supports tandem metabolic reactions linkable to oxidoreductase-based electrochemical analysis. It robustly integrates cofactors and enzymes for metabolite intermediation, detection, and interference inactivation, while self-mediating these reactions at the limit of enzyme activity. These tandem metabolic reaction–based sensors can catalyze the advancement of metabolomics from in vitro to in vivo settings, addressing missing context, real-time interaction, and high-resolution temporal dimensions in metabolomics-driven research and medical applications, such as microbiome studies and metabolic disorders.

**Fig. 1.**
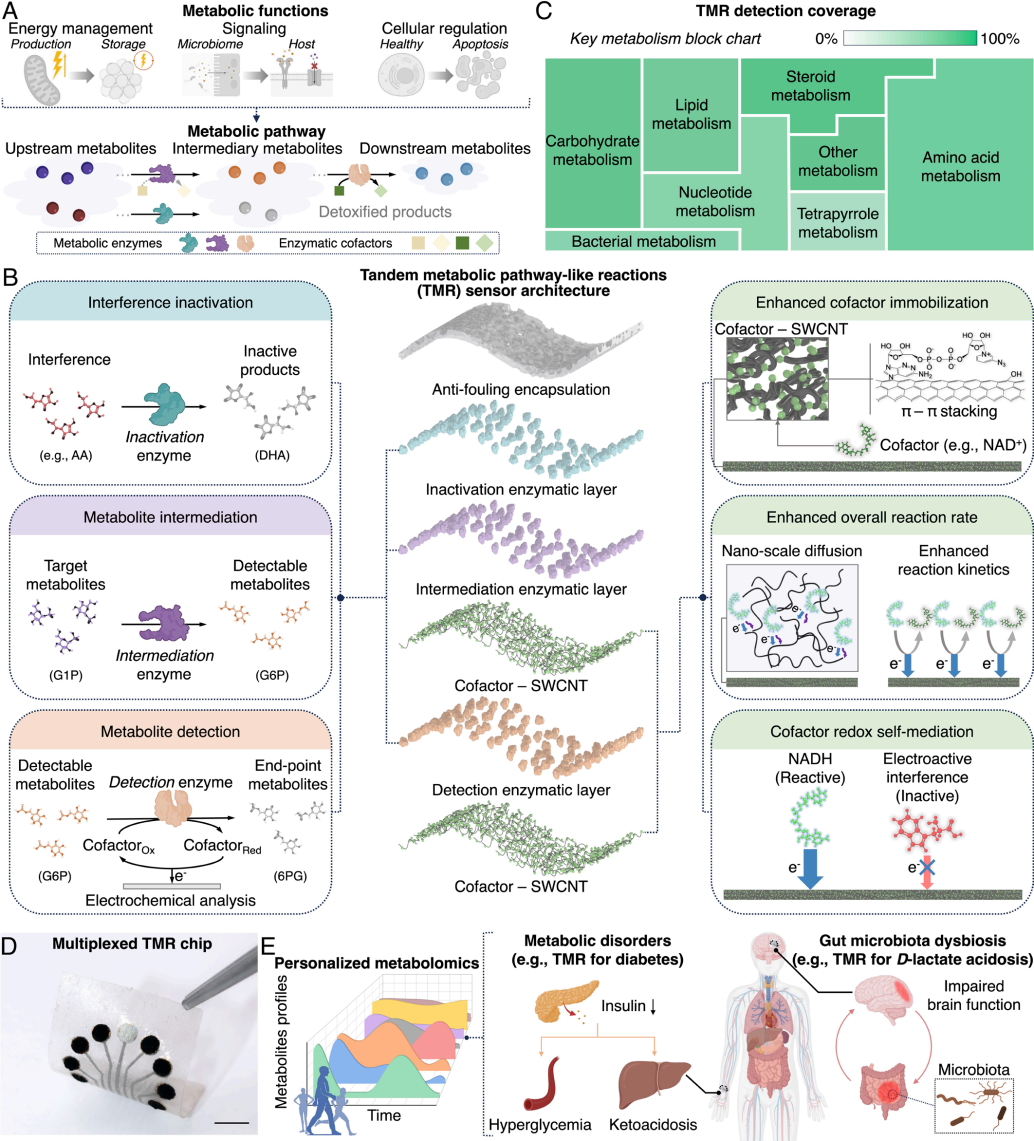
Tandem metabolic pathway–like reaction architecture for biosensing. (*A*) Schematic illustrations of example biological functions enabled by metabolites and conceptualized metabolic pathways for sequential metabolites transformation and detoxification. (*B*) Schematic illustrations of the TMR architecture (exploded view, *Middle*) with multifunctional enzymes (*Left*) and cofactors (*Right*) integrations. AA, ascorbic acid. DHA, dehydroascorbic acid. G1P, glucose 1-phosphate. G6P, glucose 6-phosphate. 6PG, 6-phosphogluconolactone. (*C*) TMR sensor design’s detection coverage across key metabolic categories. Block areas represent the number of metabolites per category, and the green gradient indicates percentage coverage (linear scale). (*D*) An optical image of a representative TMR array. (Scale bar, 5 mm.) (*E*) Schematic illustrations of the TMR enabled personalized metabolomics and diagnostics and therapeutics for human diseases.

Metabolites are small molecules that facilitate biochemical reactions necessary for sustaining life in every organism. They serve as substrates, products, or intermediates in metabolic pathways and participate in various biological processes, including energy management (production and storage), signaling, biomolecule synthesis/breakdown, and cellular regulation (Fig. 1*A*) (1, 2). These molecules can originate intrinsically from the host organism or extrinsically from various sources such as the microbiome, diet, and environmental xenobiotics (1, 3–5).

Quantifying metabolites within relevant biological contexts is essential for decoding the body’s metabolism, particularly for understanding intricate physiological systems and developing diagnostics and therapeutics. However, current metabolomic technologies, such as mass spectrometry, while capable of quantifying numerous metabolites, are limited to ex vivo analysis (3, 4, 6). This restriction results in capturing only a snapshot of the metabolome at a specific collection moment, fundamentally hindering insights into metabolites’ direct physiological roles, dynamic interconnectivity, and responses to stimuli within living organisms (1). The capital and resource intensiveness of these technologies, along with their dependence on complex sample collection and preprocessing procedures involving multiple instruments, further compromise both the sampling rate and the quality of metabolic data (3, 4, 6). All these limitations have led to persistent knowledge gaps and missed diagnostic/therapeutic opportunities in the emerging fields such as microbiome studies and personalized metabolomics (1, 7–10).

In vivo metabolite monitoring can transcend these limitations and drive the next frontier in metabolomic discoveries and healthcare. Nonetheless, current sensors offering potential for real-time and continuous measurements have limited metabolite detection coverage and encounter reliability challenges for in vivo operation—whether employing enzymatic or synthetic probes such as aptamers and molecularly imprinted polymers. Enzymatic sensors predominantly exploit single-step oxidase reactions for metabolite detection, addressing only a small subset of metabolites due to restricted reaction diversity (11, 12). The few demonstrated enzymatic metabolite sensors employing nicotinamide adenine dinucleotide (NAD, a cofactor) and dehydrogenase reactions suffer from unstable cofactor incorporation, poor reaction rates, and electroactive interference (13–17). Sensors based on synthetic probes are challenged by poor probe affinity and specificity toward metabolites, particularly when dealing with small molecules with similar physical properties (18–20). They also face stability issues due to factors like probe degradation (e.g., detachment or digestion) and susceptibility to changes in surrounding ionic strength (18, 21, 22). Additionally, extensive and uncertain discovery and engineering campaigns are required to establish synthetic probes with basic recognition properties for each metabolite (18, 23, 24).

Here, we mimic naturally refined metabolic pathways on electrodes for robust in vivo monitoring of a myriad of metabolites. To realize this strategy, we devise a single-wall carbon nanotubes (SWCNT) electrode framework that integrates multifunctional enzymes and cofactors to deliver tandem metabolic pathway-like reactions (TMR). This design provides versatility for tailoring a cascade of reactions toward end-point oxidoreductase catalysis—effectively transforming the target metabolite (the initial substrate in the cascade) into an electrochemically detectable product (Fig. 1 *B*, *Left*). In parallel, in a manner akin to detoxification in metabolism, the design can employ enzymatic inactivation to neutralize dominant interferences approaching the sensing interface, thereby enhancing the signal-to-noise ratio (SNR).

The TMR electrode offers several additional advantages for high-performance in vivo sensing (Fig. 1 *B*, *Right*). It reaches the theoretical limit of overall reaction rates set by the enzyme’s redox rate through the direct adsorption of cofactor molecules and by utilizing SWCNT’s high aspect ratio and superior electrocatalytic capabilities. The electrode’s high specific area increases the loading of enzymes and cofactors, further enhancing the SNR. Moreover, the electrode possesses self-mediating capabilities for driving cofactor oxidation at 0 V (vs. Ag/AgCl, silver/silver chloride), dramatically minimizing electrode fouling and electroactive interference. This self-mediation, in turn, obviates the need for mediators and their associated challenges (e.g., irreversible responses caused by mediator leakage) and simplifies wireless circuit requirements, aligning with envisioned in vivo operations.

Given that the vast majority of metabolites are linkable to oxidoreductase reactions via known metabolic pathways, our solution enables real-time and continuous sensing of metabolites with extremely broad coverage (12, 25). Just accounting for those linkable to oxidoreductase-based detection, either directly, or via one intermediation step, covers more than two-thirds of metabolites (Fig. 1*C* and Dataset S1). The TMR platform can easily extend to an array format for multiplexed metabolite detection by integrating the corresponding enzymes or cofactors (dictated by the target pathway) onto different TMR electrodes and employing a single shared reference electrode (Fig. 1*D*). The TMR platform could serve as a viable in vivo metabolomics tool to address the missing context, real-time interaction, and high-resolution temporal dimension in metabolomics-driven research and medical applications, such as gut–brain axis studies and the diagnosis/treatment of metabolic disorders (Fig. 1*E*).

## Results

### TMR Integrates and Self-Mediates Cofactor Redox Reactions at the Enzymatic Limit.

The electrode’s support for cofactor-assisted enzymatic reactions and electrochemical analysis of modified cofactor end-products is fundamental to versatile metabolite intermediation and detection. To put it in perspective, considering cofactor NAD alone: It facilitates roughly ten times more reactions than cofactor-less oxidase reactions, leading to a proportional increase in directly detectable metabolites (~800, Dataset S2). However, the involvement of cofactor molecules, which expands reaction versatility, also increases reaction complexity, posing two key challenges. First, the essential cofactor molecules required to drive reactions are scarce in vivo, necessitating integration into the sensor for in vivo operations (26). This contrasts with oxidase-based reactions, which benefit from naturally abundant oxygen (27). Second, the larger end-product size in cofactor-based sensing hinders the application of size-exclusion methods commonly used in oxidase sensing for interference mitigation. Coupled with high overpotential requirements, this increases the sensor’s susceptibility to interfering molecules in electrochemical analysis (27–30). The TMR electrode’s unique features overcome these challenges from multiple aspects, as we describe below.

**Fig. 2.**
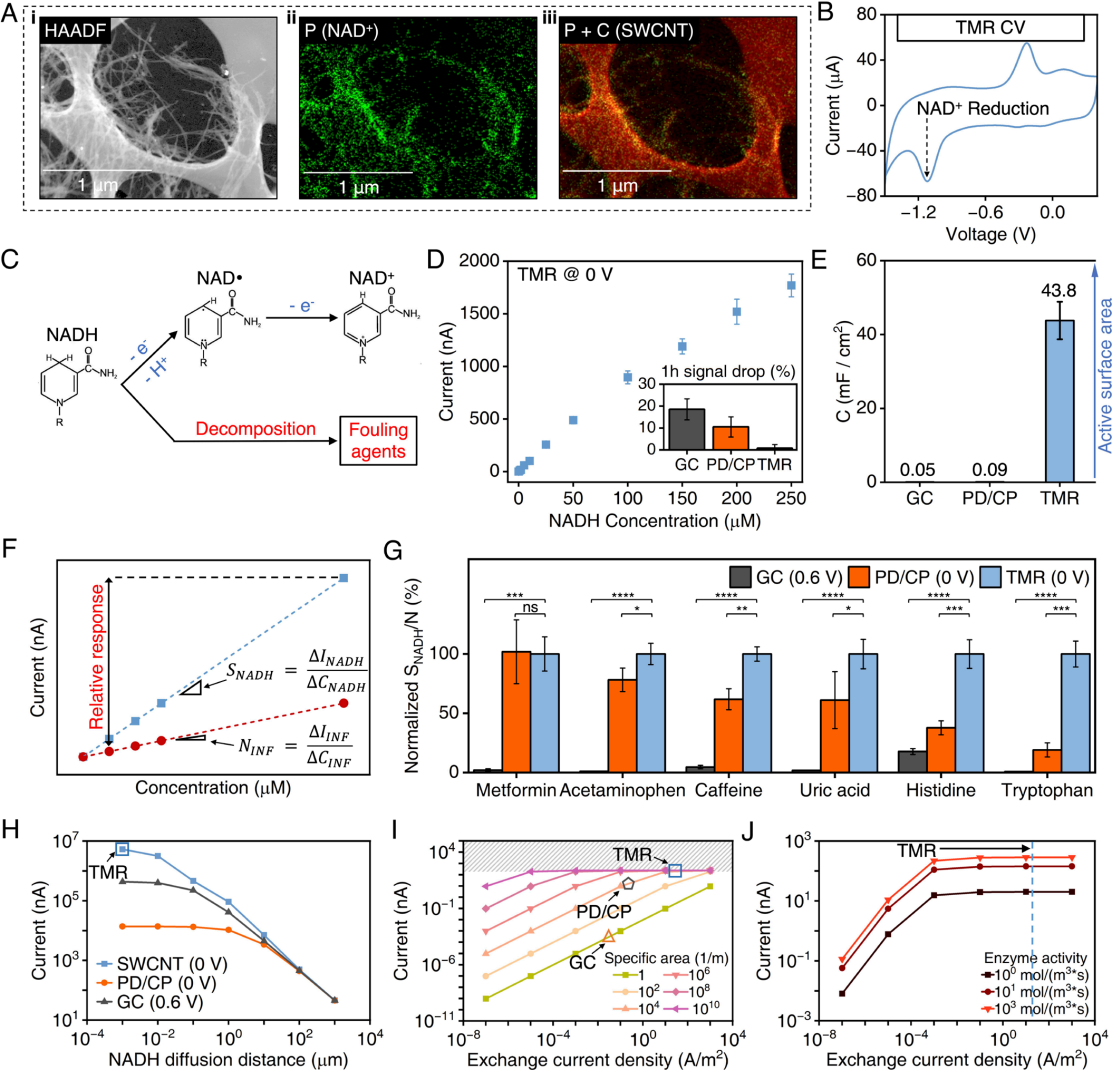
Electrochemical characterization of TMR for cofactor-assisted enzymatic reactions. (*A*) S/TEM-EDS images of TMR. HAADF, high-angle annular dark-field imaging. P, phosphorus, the signature element for NAD^+^. C, carbon. (*B*) CV characterization of TMR in PBS. (*C*) Schematic of NADH oxidation and decomposition reactions. (*D*) NADH calibration curve obtained from TMR (*N* = 3), with the inset showing the 150 μM NADH oxidation signal stability comparison with other commonly used electrodes operated at their intended applied voltages (GC at 0.6 V vs. Ag/AgCl, PD/CP at 0 V vs. Ag/AgCl). (*E*) Specific capacitance comparison among GC, PD/CP, and TMR (*N* = 3). (*F*) Schematic of sensor selectivity definitions compared between traditional relative response of analyte to interference (INF) and our SNR based on sensitivity ratio of analyte to interference. (*G*) Normalized SNR comparison among GC, PD/CP, and TMR against a panel of electroactive interferences in biofluids (*N* = 3 for each electrode, and error bars indicate SD). Statistical significance and *P* values were determined by two-tailed unpaired Student’s *t* test. ****P < 0.0001, ***P < 0.001, **P < 0.01, *P < 0.05, and “ns” denotes statistical nonsignificance. (*H*) Simulation results for 200 μM NADH oxidation currents with different diffusion distances for GC, PD/CP, and TMR. (*I*) Simulation results for 200 μM *β*-hydroxybutyrate (BHB) signal currents with different exchange current densities under different electrode-specific areas. (*J*) Simulation results for 200 μM BHB signal currents with different exchange current densities under different enzyme activities.

Cofactors such as NAD^+^ and ATP (adenosine triphosphate) can be directly adsorbed onto the electrode’s SWCNT framework through π–π stacking interactions. Utilizing scanning transmission electron microscopy (S/TEM) in conjunction with energy-dispersive X-ray spectroscopy (EDS), we visualized this integration. Fig. 2 *A*, *i* and *SI Appendix*, Fig. S1 depict the morphology of the cofactor-integrated SWCNT for the cases of NAD^+^ and ATP, respectively. The corresponding EDS images in Fig. 2 *A*, *ii* and *iii* and *SI Appendix*, Fig. S1 indicate a consistent and concentrated cofactor distribution across the SWCNT framework. The successful immobilization of NAD^+^ can be further verified via cyclic voltammetry (CV), which captures the distinctive redox signatures of this electroactive cofactor (Fig. 2*B*) (31).

The TMR electrode can electrochemically analyze the electroactive end-product of cofactor-assisted enzymatic reactions with exceptionally high efficiency. We studied its performance in the context of NAD redox reactions, chosen as a model due to their broad applicability to dehydrogenase-based systems within the oxidoreductase library. In this context, the electrochemical oxidation of NADH theoretically involves a two-electron transfer process, represented by the electron transfer number, *n_e_*, of 2 (Fig. 2*C*) (32, 33). Nonetheless, empirically, due to NADH’s inherent instability resulting in irreversible decomposition and electrode fouling, the effective electron transfer number is reduced (30, 34). For conventional electrodes such as glassy carbon (GC) and carbon paste incorporating 1,10-phenanthroline-5,6,-dione-based mediators (PD/CP), *n_e_* is close to 1 (35, 36). Our TMR electrode, based on acid-treated SWCNT, demonstrates significantly higher NADH oxidation efficiency with an *n_e_* as high as 1.90 ± 0.07 (obtained via rotating disk electrode, RDE, analysis, *SI Appendix*, Fig. S2), while also exhibiting high NADH sensitivity at 0 V (vs. Ag/AgCl) oxidation potential (i.e., self-mediating reactions, Fig. 2*D*). This enhanced performance can be attributed to the large specific area of the SWCNT substrate (Fig. 2*E* and *SI Appendix*, Fig. S3), the presence of quinone-based groups on the acid-treated SWCNT, and the catalytic property of the SWCNT’s edge plane (37, 38). The high oxidation efficiency of the TMR electrode also contributes to its superior antifouling performance. The results of our antifouling study (1-h oxidation at 150 μM NADH) illustrate that the degradation in the TMR electrode’s NADH response is less than one percent, which is over 10-fold smaller than that observed with alternative electrodes all operated at their intended voltages for NADH oxidation (Fig. 2 *D*, *Inset*). To further assess its long-term stability, the TMR sensor was subjected to continuous NADH oxidation for 10 h—10 times the duration of the initial test—where it still exhibited minimal signal degradation (1.2%, *SI Appendix*, Fig. S4).

The TMR’s self-mediation not only enhances the signal generated by electroactive end-product redox reactions but also minimizes noise from electroactive interference, thus addressing a fundamental challenge in enzymatic sensing. To illustrate this benefit, we characterized the TMR’s sensitivity to NADH and a panel of electroactive molecules commonly found in biofluids (39). For comparison, we conducted the same procedure using GC and PD/CP electrodes and defined the ratio of the electrodes’ reaction sensitivity to NADH vs. interfering analytes as a measure of SNR (Fig. 2*F*). This definition of SNR offers comprehensive coverage across a spectrum of target and interference concentrations, ensuring applicability to diverse sensing scenarios. It is distinct from traditional single-point interference characterization methods, which often use large concentration differences between the target and interfering molecules (16). Fig. 2*G* demonstrates that for all interference cases, the TMR exhibited significantly higher SNR compared to the alternatives (*SI Appendix*, Fig. S5).

The TMR architecture also enhances both the mass transport of the enzymatic reaction product and the associated electrochemical reaction kinetics, ultimately achieving optimal overall reaction rates. In TMR, cofactor molecules (NAD^+^) are directly immobilized onto the porous electrode substrate with a large specific area (*A_0,TMR_* ~ 10^8^/m). This design reduces the diffusion distance of the enzyme product (NADH) to the substrate electrode, down to a few nanometers. In contrast, conventional cofactor-based enzymatic sensors superficially immobilize cofactors atop the electrode substrate, such as GC and PD/CP, within the enzyme layer of approximately a few micrometers thickness (15, 40). Consequently, their diffusion length scales are on the order of micrometer scale. Furthermore, the TMR drives NADH oxidation at a substantially higher intrinsic rate than conventional electrodes, as evidenced by its large exchange current density (*i_0,TMR_* ~ 17.4 ± 0.3 A/m^2^, *SI Appendix*, Fig. S2, as compared to *i_0,GC_* ~ 0.06 A/m^2^ and *i_0,PD/CP_* ~ 0.35 A/m^2^) (35, 41). This together with TMR’s extremely large specific area (Fig. 2*E* and *SI Appendix*, Fig. S3), allows TMR to drive overall catalytic reactions (within a fixed footprint) at a dramatically higher capacity than alternative electrodes.

To study the TMR’s enhanced enzymatic/electrochemical reaction kinetics, we utilized a finite element analysis–based simulation model. Within this model, key parameters such as cofactor arrangement, enzyme activity, and electrode properties/design (e.g., *i_0_*, *A_0_*), can be explored. We first validated the fidelity of the model by verifying its alignment with empirical measurements (*SI Appendix*, Fig. S6). Then, to study the effect of mass transport limitation, we simulated the transduced NADH oxidation current resulting from different NADH diffusion distances across three types of electrodes: SWCNT (TMR’s framework), GC, and PD/CP (operated at their intended oxidation voltages) (32). As shown in Fig. 2*H*, the results indicate that TMR transduces over 100-fold larger current. Next, we studied the reaction kinetics of the electrodes in terms of their specific area and exchange current density. To conduct this study in isolation from mass transport limitations, we reconfigured the model to simulate NADH oxidation (generated by a model dehydrogenase reaction) directly taking place at the electrodes’ surfaces. The corresponding simulation results for a range of hypothetical exchange current densities and specific areas are shown in Fig. 2*I*. They specifically indicate that not only does the TMR substantially outperform alternative GC and PD/CP electrodes, but it also facilitates catalytic reaction at a rate only limited by the enzyme activity (as evident from plateaued current response). Reaching this limit can be achieved across a wide range of enzyme activities, as shown in Fig. 2*J*.

### TMR Integrates Multifunctional Enzymatic Reactions for Versatile Metabolite Monitoring.

Besides its distinguishing support for cofactor-assisted enzymatic/electrochemical reactions, the TMR platform can facilitate multiple enzymatic reactions in tandem, further setting it apart from conventional in vivo enzymatic sensors designed for single reactions. We harnessed these special properties for direct/intermediated metabolite detection and supplemental interference inactivation (further enhancing SNR in our system).

**Fig. 3.**
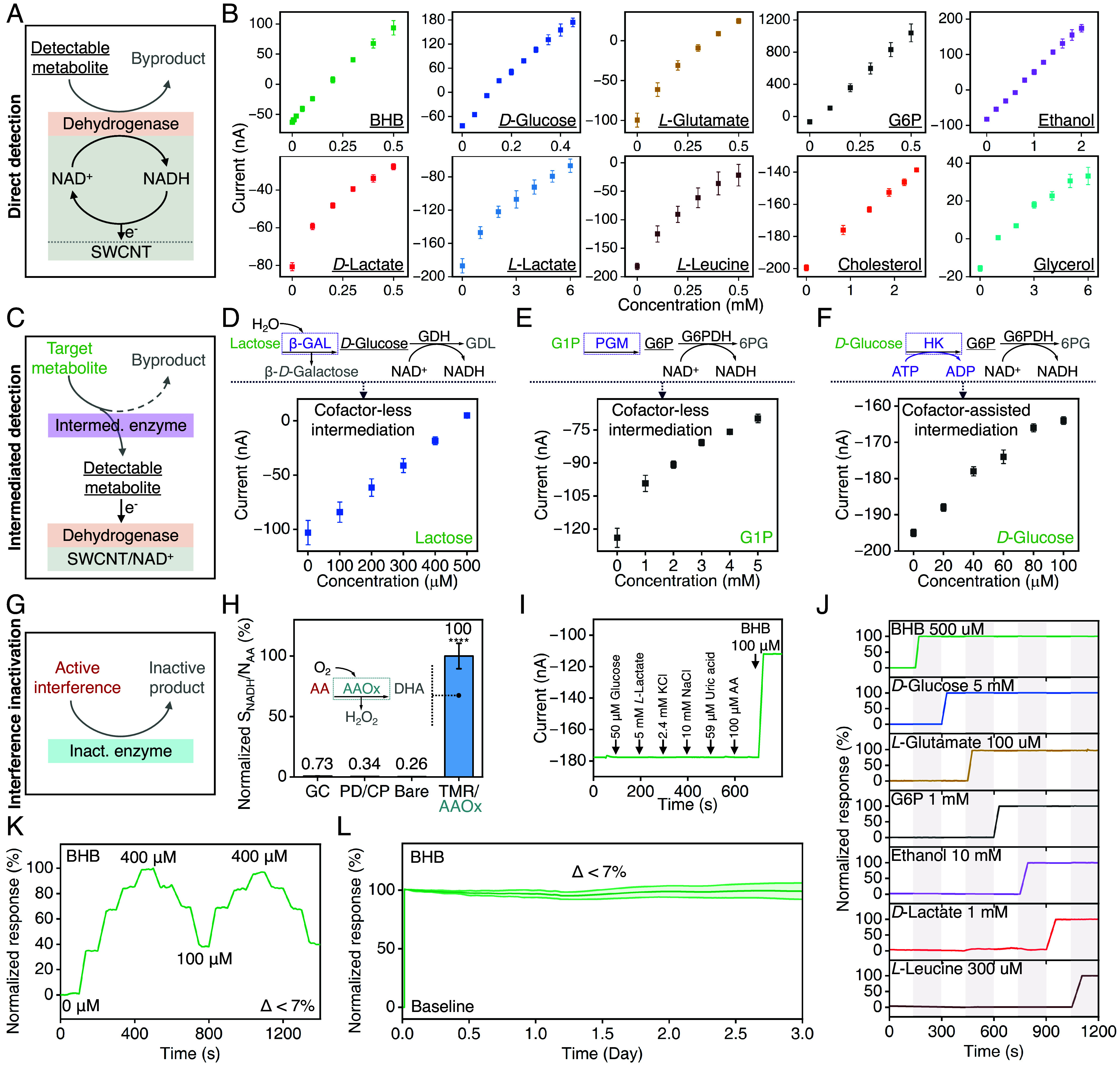
Characterization of TMR metabolite sensors. (*A*) Schematic of TMR-based direct metabolite detection. (*B*) TMR calibration responses targeting BHB, *D*-glucose, *L*-glutamate, G6P, ethanol, *D*-lactate, *L*-lactate, *L*-leucine, cholesterol, and glycerol. *N* = 3 for each tested TMR, and error bars indicate SD. (*C*) Schematic of TMR-based intermediated metabolite detection. (*D*–*F*) TMRs’ intermediated detection mechanism and calibration responses targeting lactose (*D*), G1P (*E*), and *D*-glucose (*F*). *N* = 3 for each tested TMR, and error bars indicate SD. GDH, glucose dehydrogenase. GDL, *D*-Gluconic acid δ-lactone. G6PDH, G6P dehydrogenase. HK, hexokinase. ADP, adenosine diphosphate. (*G*) Schematic of TMR-based interference inactivation. (*H*) Normalized SNR comparison among GC, PD/CP, bare TMR, and AAOx-coupled-TMR against AA. *N* = 3 for each tested sensor, and error bars indicate SD. (*I*) Real-time amperometric selectivity study with a representative BHB-TMR. (*J*) Multiplexed real-time amperometric measurements in undiluted porcine serum with a panel of TMRs targeting BHB, *D*-glucose, *L*-glutamate, G6P, ethanol, *D*-lactate, and *L*-leucine. (*K*) Real-time BHB amperometric responses with a representative BHB-TMR. (*L*) A 3-d long real-time amperometric measurement performed with BHB-TMRs in PBS supplemented with a 2 mM BHB increase from baseline (0 mM). Normalized response = (*I* – *I_Baseline_*)/(*I_Max_* – *I_Baseline_*), error band indicates SD (*N* = 3).

To demonstrate direct metabolite detection, we employed dehydrogenase enzymes along with the cofactor NAD^+^ to catalyze metabolic reactions, resulting in NADH as the electroactive end-product (Fig. 3*A*). In this setting, metabolite substrates, serving as targets, are quantified through the electrochemical analysis of NADH (self-mediated by SWCNT). We introduced ten different dehydrogenases into the TMR design, each targeting a distinct metabolite: *β*-hydroxybutyrate (BHB), *D*-glucose, *L*-glutamate, glucose 6-phosphate (G6P), ethanol, *D*-lactate, *L*-lactate, *L*-leucine, cholesterol, and glycerol. Calibration plots for each enzymatic TMR were obtained through amperometric measurements conducted within the physiological concentration ranges of the analytes (as detailed in *SI Appendix*, Table S1). Fig. 3*B* shows that all TMR sensors exhibited consistent, monotonic responses to target metabolite concentrations with reproducible sensitivities and minimal interdevice variations.

To target metabolites lacking specific oxidoreductases, we utilized existing metabolic pathways and harnessed the TMR’s versatility to build an oxidoreductase linkage. We configured the TMR to facilitate cascaded enzymatic reactions, intermediating the target metabolite into a form catalyzable by a corresponding oxidoreductase enzyme for electrochemical detection (Fig. 3*C*). We demonstrated both cofactor-less and cofactor-assisted enzymatic intermediation by integrating appropriate enzymes and, when necessary, cofactors within the TMR architecture. We leveraged the dehydrogenase-based electrochemical sensing interfaces developed for direct detection as the TMR’s base to facilitate the final stage of the cascade. This integrated design implements the entire intermediation and detection stages within a single sensor construct.

With cofactor-less intermediation, we demonstrated the sensing of lactose and glucose 1-phosphate (G1P), which lack corresponding oxidoreductases. By integrating *β*-galactosidase (*β*-GAL) and phosphoglucomutase (PGM) enzymes within the original *D*-glucose-TMR and G6P-TMR sensing interfaces, we transformed lactose and G1P into *D*-glucose and G6P for subsequent electrochemical detection, respectively (Fig. 3 *D* and *E*). To illustrate the extensibility of our approach to cofactor-assisted intermediation, we drew inspiration from the glycolysis pathway and demonstrated intermediated glucose detection as proof of concept (42). Using hexokinase and ATP cofactors integrated within the TMR framework, *D*-glucose is converted into G6P, whose concentration is measurable via the NAD^+^/G6P-dehydrogenase base (Fig. 3*F*). As shown in Fig. 3 *D*–*F*, all TMR sensors implementing cascaded reactions consistently displayed monotonic responses to varying concentrations of target metabolites, exhibiting reproducible sensitivities and minimal variations. In the case of severe fluctuations in intermediary metabolite levels in vivo affecting the sensor response, the confounding effect can be mitigated by calibrating the primary TMR sensor response against a secondary TMR detecting the intermediary metabolites. Since the primary TMR incorporates the design of the secondary TMR as its subunit, accurate concentration estimation is ensured (*SI Appendix*, Fig. S7).

We specifically employed enzymatic inactivation to neutralize interference from ascorbic acid (AA) by integrating the corresponding enzyme layer (AA oxidase, AAOx). In most enzymatic sensing scenarios, including cofactor-based ones, AA serves as the most dominant noise source, distorting sensor responses through unwanted reactions with the electrodes’ substrates (Fig. 3*G*) (11, 13, 16, 17, 29, 43). The TMR’s exceptionally large specific area makes it suitable for immobilizing AAOx with high loading to effectively counter this challenge. We followed the aforementioned procedure for characterizing the SNR to study and benchmark the performance of the AAOx-coupled TMR in minimizing interference from AA. Fig. 3*H* shows that our enzymatic inactivation strategy was extremely effective, as evidenced by the AAOx-coupled TMR’s more than 100-fold larger SNR compared to other traditionally used electrodes (*SI Appendix*, Fig. S8). To further ensure the TMR’s selectivity, we recorded the representative BHB, *D*-glucose, and *L*-glutamate-TMRs’ responses to a panel of progressively introduced molecules, including small molecules, ionic species, and electroactive species at their physiologically relevant concentrations, with AA (dominant interference) tested at a high concentration (100 μM, compared to 50 μM, high end of salivary AA concentration) (44). As shown in Fig. 3*I* and *SI Appendix*, Fig. S9, the TMRs exhibited negligible response against the interference group or the enzymatic inactivation product (here, hydrogen peroxide generated by the AAOx-catalyzed reaction) due to its self-mediating capability for driving cofactor oxidation at 0 V. The latter findings particularly illustrate there is no reaction crosstalk between the two enzymatic layers (i.e. interference inactivation and detection enzymes).

To demonstrate multiplexed metabolite monitoring, we fabricated an array of 7 TMRs onto a soft substrate (styrene–ethylene–butylene–styrene block copolymer, SEBS), sharing a single reference electrode. Each TMR targeted a specific metabolite: BHB, *D*-glucose, *L*-glutamate, G6P, ethanol, *D*-lactate, and *L*-leucine. We tested this array’s response in serum by concurrently recording the amperometric measurements of all 7 channels and intermittently introducing individual analyte targets. As shown in Fig. 3*J*, the sensors stably responded to their corresponding analytes with no detectable crosstalk.

The incorporation of an encapsulation layer (here, polyvinyl chloride, PVC), within the TMR design, combined with the robust immobilization of cofactor molecules on the TMR’s framework, ensures the stability and reversibility of the enzymatic TMR’s response. The reversibility of TMR was assessed by repeatedly immersing representative BHB, *D*-glucose, and *L*-glutamate-TMRs in solutions with increasing or decreasing target concentrations and continuously recording their responses at each concentration level. In all cases, the TMRs consistently adjusted to the expected response levels, with changes of less than 7.5% for each introduced concentration (Fig. 3*K* and *SI Appendix*, Fig. S10). The TMRs’ antifouling capability was assessed through continuous measurements in a protein-rich environment (phosphate-buffered saline, PBS, buffer with 20 mg/mL bovine serum albumin) (45). During 1,000-min studies involving varying target concentrations, the enzymatic TMR responses’ declines were within 2% at each level (*SI Appendix*, Fig. S11), demonstrating the TMR’s ability to facilitate small molecule (i.e., metabolite) diffusion to the sensing substrate while effectively blocking larger protein molecules (fouling agents). We also conducted a prolonged characterization study, continuously recording the TMR’s response in a PBS buffer. As shown in Fig. 3*L*, the TMR exhibited minimal response deviation, remaining within a few percentages, even after 3 d of continuous operation, indicating negligible leakage of sensing molecules.

### TMR Tracks Metabolite Dynamics In Vivo for Metabolic Disorders and Gut–Brain Axis.

Collectively, the ex vivo characterization results support the high level of adaptability, sensitivity, selectivity, stability, and reversibility of the TMR architecture for in vivo biomonitoring. After validating the TMR’s biocompatibility through cellular viability studies (*SI Appendix*, Fig. S12), we adapted and deployed the TMR sensors for two metabolic acidosis scenarios: ketoacidosis and *D*-lactate acidosis. For both scenarios, we initially established the significance of the target metabolites in relevant biomatrices and evaluated the accuracy of the TMR sensors for their analysis. Then, we applied the TMR sensors for real-time and continuous in vivo monitoring, demonstrating their potential for tracking the metabolite dynamics underlying metabolic states and the gut–brain axis.

**Fig. 4.**
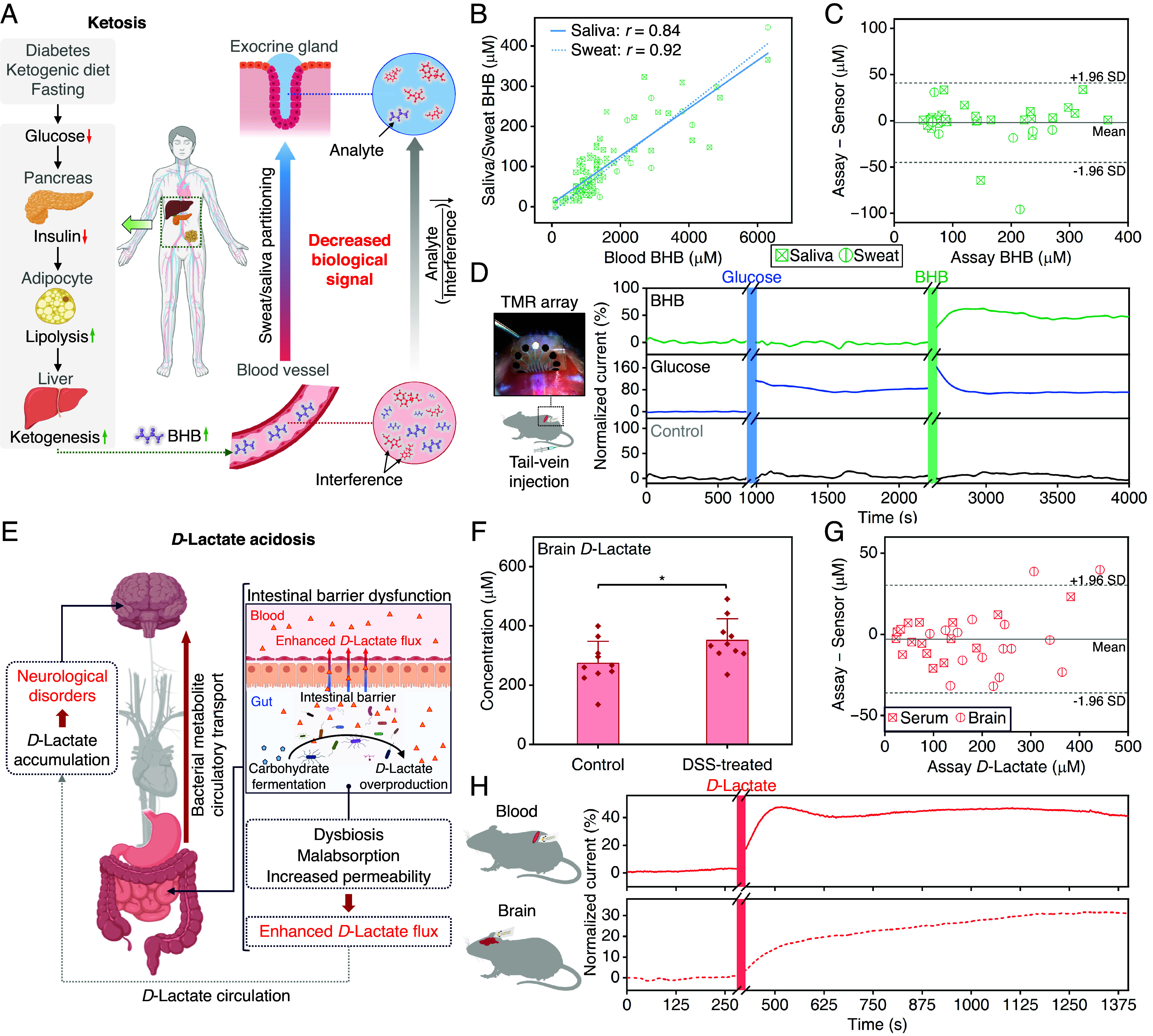
Metabolic acidosis studies with TMRs. (*A*) Schematic illustrations of ketosis mechanism and diffusion of metabolites from blood to noninvasively retrievable biofluids (e.g., sweat and saliva) in the human body. (*B*) Saliva–blood and sweat–blood BHB concentration correlations for healthy subjects with a ketone supplement and epileptic patients under ketogenic diets. (*C*) Bland–Altman plot comparing the standard assay-quantified BHB concentrations with the TMR-measured BHB concentrations in human saliva and sweat samples. (*D*) In vivo real-time blood BHB and glucose monitoring in a mouse with multiplexed TMRs functionalized for BHB, glucose, and negative control. The shaded green and blue bands indicate the BHB and glucose tail-vein injection time windows, respectively. The inset shows the experimental setup. Normalized current = (*I* – *I_Baseline_*)/*I_Baseline_*. (*E*) Schematic illustration of *D*-lactate acidosis. (*F*) *D*-lactate concentrations in brain samples from control vs. DSS-treated mice. Statistical significance and *P* value were determined by two-tailed unpaired Student’s *t* test. * *P* < 0.05. (*G*) Bland–Altman plot comparing the standard assay-quantified *D*-lactate concentrations with the TMR-measured *D*-lactate concentrations in mice serum and brain samples. (*H*) In vivo real-time blood and brain *D*-lactate monitoring with TMRs in a rat model. The shaded bands indicate the *D*-lactate tail-vein injection time windows. The inset shows the experimental setup.

Ketoacidosis, a serious metabolic disorder often associated with diabetes, arises when ketone bodies like BHB accumulate in the bloodstream, causing an acid–base imbalance (46). Here, we first investigated the utility of BHB sensing in sweat and saliva for noninvasive wearable and mobile health monitoring. This approach is beneficial for individuals with conditions such as diabetes or those on ketogenic diets for epilepsy (46, 47). However, from a sensing perspective, it is challenging due to over 10-fold secretion-induced dilution of BHB and high background noise from fluctuating interfering molecules such as AA (often influenced by diet), which current enzymatic electrochemical sensors fail to address (Fig. 4*A*) (16, 17, 44, 48, 49). Our TMR sensor, with its intrinsically high SNR measurements and low limit of detection (*SI Appendix*, Fig. S13), can effectively overcome these challenges. We sampled saliva and sweat from two cohorts: epileptic patients on a ketogenic diet and healthy subjects who consumed a ketone supplement. Our results showed strong correlations between sweat and saliva BHB levels vs. blood (*r* = 0.84 for saliva–blood, and *r* = 0.92 for sweat–blood, Fig. 4*B*), also validating our BHB-TMR’s accuracy in analyzing sweat and saliva (mean bias −2 μM with 95% CI within ±45 μM, Fig. 4*C*). We confirmed the TMR’s compatibility with low-power consumer electronics for wireless operation (*SI Appendix*, Fig. S14) and used it to track changes in metabolic states. *SI Appendix*, Fig. S15 depicts the TMR detecting elevated salivary BHB concentration in a fasting healthy subject, indicating ketosis. Following consumption of a carbohydrate-rich beverage, salivary BHB levels rapidly dropped from ~250 to ~100 μM within an hour, suggesting a shift from ketosis to glycolysis (corroborated by capillary blood glucose analysis) (50).

To validate TMR’s in vivo monitoring capability relevant to diabetic ketoacidosis, we tracked blood BHB and glucose levels in mice. BHB- and glucose-TMRs were integrated into an array, alongside a TMR lacking detection enzymes serving as a negative control. As shown in Fig. 4*D* and *SI Appendix*, Fig. S16, following intravenous administration of each metabolite, both BHB and glucose TMRs (affixed on the mouse back for subdermal blood analysis) promptly captured the dynamic changes of the corresponding analytes, while the negative control maintained its baseline response. The results highlight the TMR’s ability for in vivo metabolic data acquisition with minute-level resolution, surpassing the sampling rates of traditional methods by two to three orders of magnitude, especially advantageous in small animals with limited sampling volume thresholds (51).

For *D*-lactate acidosis, we focused on detecting the bacterial metabolite *D*-lactate in blood and brain for its applications in disease diagnosis and treatment (e.g., short bowel syndrome and encephalopathy) and for advancing understanding of microbiome–gut–brain axis dynamics.

*D*-lactate is a byproduct of gut bacterial carbohydrate fermentation (Fig. 4*E*). Several interacting factors, including dysbiosis of the gut microbiota, malabsorption of intestinal nutrients, and decreased intestinal barrier integrity can enhance flux of *D*-lactate from the intestinal lumen into systemic circulation (52). *D*-lactate typically crosses the blood–brain barrier via monocarboxylate transporters, but can exhibit increased translocation in pathological conditions. Within the brain, *D*-lactate accumulates at least in part due to its slower metabolism compared to endogenous *L*-lactate, leading to neurotoxicity and various neurological complications, including confusion, disorientation, and seizures (52).

To study the effect of bacterial fermentation on *D*-lactate levels in blood vs. brain, we monocolonized mice with *Bacteroides thetaiotaomicron*, a prominent member of the human gut microbiome that plays a crucial role in digesting complex carbohydrates. Mice were then fed a custom diet containing the host nondigestible carbohydrate levan as the sole carbohydrate source, which *B. thetaiotaomicron* selectively ferments (53). *SI Appendix*, Fig. S17 shows that, compared to germ-free controls, colonization with *B. thetaiotaomicron* modestly increased serum *D*-lactate levels without elevating brain *D*-lactate levels. This suggests that under nonpathological conditions, the gut microbiome promotes *D*-lactate in the serum without affecting brain levels. We next modeled high carbohydrate feeding and intestinal barrier dysfunction as key risk factors for *D*-lactate acidosis and comorbid encephalopathy. To do so, we first treated conventionally colonized mice with dextran sodium sulfate (DSS), a common model of experimental colitis, to induce intestinal barrier permeability (54). Following 7 d of DSS treatment, mice were fasted and orally gavaged with a mixture of host nondigestible carbohydrates (fructooligosaccharide, inulin, cellulose, and gum arabic) to promote rapid bacterial fermentation and *D*-lactate production. Fig. 4*F* demonstrates that DSS-treated mice exhibited significantly elevated *D*-lactate levels in the brain compared to vehicle-treated controls, indicating that intestinal injury leads to increased entry of bacterial-derived *D*-lactate into the brain.

There was no significant correlation between serum and brain *D*-lactate levels within individual animals (*SI Appendix*, Fig. S18), emphasizing the necessity for methods that can concurrently measure bacterial metabolites in circulation and local environments like the brain to better understand the microbiota–host interactions. The TMR proves to be a fitting solution, given its high accuracy in analyzing *D*-lactate in both blood and brain matrices, as demonstrated in Fig. 4*G* (mean bias −3 μM with 95% CI within ± 36 μM).

We deployed TMR sensors for in vivo monitoring of local and circulating *D*-lactate in a rat model. We affixed *D*-lactate-TMRs to the brain and back for subdural and subdermal analysis, respectively. An accompanying *L*-lactate-TMR sensor analyzing blood served as a negative control. We continuously recorded the TMR sensor responses before and after intravenous *D*-lactate injection. The control device exhibited minor transient disturbances postinjection (*SI Appendix*, Fig. S19), attributable to the momentarily increased osmotic load from the lactate buffer injection, consistent with prior reports (55). As shown in Fig. 4*H*, the *D*-lactate sensors tracked acute increases in *D*-lactate following the injection, revealing a slower rate of concentration increase in the brain compared to the blood, suggesting limited transport rates of *D*-lactate into the brain (56).

## Discussion

Our strategy harnesses naturally proven metabolic pathways that are linkable to oxidoreductase-based electrochemical analysis as a blueprint for bioelectronic design. Implemented through the TMR electrode with exceptional electrochemical properties, this design makes multifunctional use of evolutionarily robust molecular toolkits (enzymes and cofactors) to support underlying reactions. This approach enables reliable monitoring of a plethora of metabolites, with NAD-assisted enzymatic sensing alone capable of directly detecting over 800 metabolites.

To support broader in vivo applications, TMR sensors could benefit from enhancing their antifouling properties (e.g., exploring the use of surfactants) (57), further miniaturization, and integration with soft or microneedle bioelectronic substrates (58) or lateral flow devices for analyzing various biomatrices in diverse clinical settings. Additionally, the TMR’s solution-based fabrication is compatible with industrial manufacturing processes, enabling flexible and streamlined large-scale production.

TMR’s versatility in monitoring endogenous and bacterial metabolites in vivo across various biomatrices makes it a powerful metabolomics tool for propelling biomedical research and healthcare. In microbiome research, it can help decipher the temporal dynamics of microbiota–host metabolic communication, recognized as one of the “greatest challenges” in the field (7). TMR’s adaptation into wearable and implantable formats can advance sparse metabolite-based point-of-care testing to continuous point-of-person monitoring for chronic disease prevention/management, fitness optimization, and infectious disease detection (59). Moreover, TMR’s focus on metabolic pathways aligns seamlessly with tracking bacterial and tumor metabolism within their microenvironments (60). This capability can empower the design and monitoring of the efficacy of antibiotics and chemotherapeutics targeting key metabolic processes within pathogens or cancer cells to minimize drug resistance (61). Thus, future efforts should also include large-scale clinical trials to validate TMR’s clinical utility in these applications, while ensuring adherence to regulatory standards for safe and effective implementation.

Ultimately, scaling and deployment of TMR in these contexts will generate massive, multidimensional, and real-time metabolic datasets with high temporal resolutions, facilitating deeper understanding and interaction with biology and advancing personalized medicine.

## Materials and Methods

A detailed description of the materials and methods used in this study can be found in *SI Appendix*, including information on the fabrication and characterization of our TMR-based sensors, the electrochemical reaction simulation models, biocompatibility tests, biological sample collection and quantification, the design and operation of the wireless printed circuit board (PCB) module, and in vivo animal and human subject studies. All animal experiments were performed in compliance with protocols approved by the University of California, Los Angeles Animal Research Committee (UCLA ARC Protocol Nos. 2015-079, 2019-019, and 2021-011). The conducted human subject experiments were performed in compliance with the protocols that are approved by the Institutional Review Board at the University of California, Los Angeles (IRB#17-000170). All subjects gave written informed consent before participation in the study.

## Supplementary Material

Appendix 01 (PDF)

Dataset S01 (XLSX)

Dataset S02 (XLSX)

## Data Availability

All study data are included in the article and/or supporting information.
