# Supplementary material for: Tandem metabolic reaction–based sensors unlock in vivo metabolomics
Source: Proc Natl Acad Sci U S A. 2025 Feb 27;122(9):e2425526122. doi: 10.1073/pnas.2425526122 (PMC11892595; doi:10.1073/pnas.2425526122)
Supplement: Supplementary file 1 — Appendix 01 (PDF) [file pnas.2425526122.sapp.pdf]

PNAS

Supporting Information for
Tandem Metabolic Reaction-based Sensors Unlock In-vivo Metabolomics

Xuanbing Cheng, Zongqi Li, Jialun Zhu, Jingyu Wang, Ruyi Huang, Lewis W. Yu, Shuyu Lin, Sarah Forman, Evelina Gromilina, Sameera Puri, Pritesh Patel, Mohammadreza Bahramian, Jiawei Tan, Hannaneh Hojaiji, David Jelinek, Laurent Voisin, Kristie B. Yu, Ao Zhang, Connie Ho, Lei Lei, Hilary A. Coller, Elaine Y. Hsiao, Beck L. Reyes, Joyce H. Matsumoto, Daniel C. Lu, Chong Liu, Carlos Milla, Ronald W. Davis, and Sam Emaminejad

Corresponding authors: Ronald W. Davis, Sam Emaminejad
Email: katrina.hong@stanford.edu, emaminejad@ucla.edu

This PDF file includes:

Supporting text
Figures S1 to S19
Table S1
Legends for Datasets S1 to S2
SI References

Other supporting materials for this manuscript include the following:

Datasets S1 to S2

Supporting text

Materials. Single-walled carbon nanotubes (SWCNT), reduced nicotinamide adenine dinucleotide (NADH), nicotinamide adenine dinucleotide (NAD^+), metformin, uric acid (UA), caffeine, acetaminophen (APAP), *L*-histidine (HIS), *L*-tryptophan (TRY), *L*-ascorbic acid (AA), *DL-β*-hydroxybutyric acid sodium salt (BHB), *D*-(+)-glucose, *L*-glutamic acid, *D*-glucose 6-phosphate sodium salt (G6P), α-*D*-glucose *1*-phosphate disodium salt hydrate (G1P), *L*-leucine, glycerol, sodium *D*-lactate, sodium *L*-lactate, lactose, adenosine 5' -triphosphate disodium salt hydrate (ATP) , potassium chloride (KCl), sodium chloride (NaCl), silver nitrate ($AgNO_3$), bovine serum albumin (BSA), glutaraldehyde solution (25 wt%), polyvinyl chloride (PVC), tetrahydrofuran (THF), ethyl alcohol, *β*-galactosidase, hexokinase, phosphoglucomutase (PGM), *L*-lactate dehydrogenase, alcohol dehydrogenase, and deproteinizing sample preparation kit were purchased from Sigma-Aldrich (MO, USA). *D*-3-hydroxybutyrate dehydrogenase, glucose dehydrogenase, glutamate dehydrogenase, glucose-6-phosphate dehydrogenase (G6PDH), *D*-lactate dehydrogenase, cholesterol dehydrogenase, leucine dehydrogenase, glycerol dehydrogenase, and ascorbate oxidase were purchased from Toyobo USA, Inc. (NY, USA). Water (Optima LC–MS Grade), toluene, sulfuric acid (98%), nitric acid (70%), porcine serum, tissue protein extraction reagent, and phosphate-buffered saline (1×, Gibco PBS, pH 7.2) were purchased from Fisher Scientific (MA, USA). Sliver/silver chloride (Ag/AgCl) ink was purchased from Ercon Incorporated (MA, USA). Glassy carbon electrode (GC, diameter: 3.0 mm) and disc gold electrode (diameter: 1.6 mm) were purchased from Bioanalytical Systems Inc. (IN, USA). Mediator modified screen-printed carbon electrodes (110PHEN) were purchased from Metrohm AG (Switzerland). PILOGEL discs were purchased from ELITechGroup Inc. (UT, USA). Polyethylene terephthalate (PET, 100 µm thick) was purchased from MG Chemicals (BC, Canada). Styrene–ethylene–butylene–styrene block (SEBS, H1062) was purchased from Asahi Kasei (Japan). Double-sided tape (170 µm thick, 9474LE 300 LSE), anisotropic conductive film tape (ACF, 9703), and Tegaderm film were purchased from 3M Science (MN, USA).

Construction of the SWCNT-based NADH sensor and tandem metabolic pathway-like reactions-based sensors (TMR). The biosensors were fabricated on gold (Au) electrodes (diameter: 3 mm; 30 nm chromium, Cr/100 nm Au), deposited and patterned on a PET substrate or SEBS substrate. The SEBS substrate was fabricated by dissolving SEBS blocks in toluene (150 mg/mL) and vortexed overnight. Then the SEBS solution was drop-cast on the silver trace and cured at 90 °C for 2 hours, followed by the attachment of ACF tape. The reference electrode was fabricated by depositing 3.5 µL of Ag/AgCl ink on the Au electrode, then dried on a hot plate at 70 °C for 20 min.

An acid-treated SWCNT solution (12 mg/mL in PBS) was prepared by dispersing SWCNT into a mixture of sulfuric acid and nitric acid solution (1:3) and heating at 80 °C for 4 hours while stirring followed by centrifuging and washing with PBS to remove the residual acid.

For NADH sensors, 3.5 µL of the acid-treated SWCNT solution was drop-cast onto the Au electrode and dried in the ambient environment.

For enzymatic TMRs, NAD^+ was integrated into SWCNT solution (12 mg/mL in PBS) by dispersing acid-treated SWCNT into a NAD^+ solution (50 mM in PBS) and stirring at 4 °C for 20 hours followed by centrifuging and washing with PBS and water. 3.5 µL of the NAD^+ integrated SWCNT solution was drop-cast onto the Au electrode and dried in the ambient environment. Then, the electrode was further functionalized with a dehydrogenase layer (DH) by drop-casting an enzyme solution, which was prepared following the instruction provided by the vendor. To develop the BHB sensor, 1.13 µL of the BHB dehydrogenase solution (1056 U/mL) was drop-cast onto the Au/SWCNT electrode and dried in the ambient environment. Then, another 1.75 µL of the NAD^+ integrated SWCNT was drop-cast onto the Au/SWCNT-NAD^+/DH electrode and dried in the ambient environment. The integration of ATP into SWCNT and the fabrication of other enzymatic TMRs follows the same procedure described above with the use of their corresponding enzyme solution (800 U/mL for glutamate dehydrogenase, 20000 U/mL for G6P dehydrogenase, 5000 U/mL for leucine dehydrogenase, and 8000 U/mL for the rest of the enzymes). For sensors with intermediation functions, 1.13 µL of intermediation enzyme solution (5000 U/mL *β*-galactosidase, PGM, and hexokinase) was drop-cast onto the Au/SWCNT-NAD^+/DH/SWCNT-NAD^+ (ATP) electrode. To eliminate the interference from AA, 1.13 µL of the ascorbate oxidase solution (15000

U/mL) was drop-cast onto the Au/SWCNT-NAD$^+$/DH/SWCNT-NAD$^+$ electrode, followed by drop-casting 1 µL of the glutaraldehyde solution (0.4 wt% in PBS solution). An anti-fouling encapsulation layer was deposited by drop-casting of 1 µL of the PVC solution (0.1 wt% in THF) for 3 times. Sensors were allowed to dry overnight at 4 °C, while being protected from light. The sensors were stored at 4 °C in the dark when not in use.

Electrochemical characterization of NADH sensors. Amperometric measurements: the calibration curves were measured in a continuously stirred PBS buffer (1× PBS) at +0 V (vs. Ag/AgCl). All electrochemical characterizations were performed by a potentiostat (CHI 1040C, CH Instruments, Inc.). By stepwise addition of increasing concentrations of the NADH stock solution in the PBS buffer, a calibration plot was obtained. Sensitivity was calculated as the slope divided by the sensor's surface area. The signal degradation of GC, 1,10-phenanthroline-5,6-dione/carbon paste (PD/CP) and SWCNT-based NADH sensor were measured by the percentage of current drop in a 150 µM NADH solution in the PBS buffer after 1-hour amperometric measurement at 0.6 V (vs. Ag/AgCl for GC) or 0 V (vs. Ag/AgCl for PD/CP and SWCNT). The signal-to-noise ratio (SNR) was calculated by the ratio of the sensitivity of analyte to the sensitivity of the interference. The sensitivity of the interference was characterized by stepwise addition of various interferences into the PBS, including metformin, UA, caffeine, APAP, HIS, TRY, and AA.

Rotating disk electrode linear scan voltammetry (LSV): the electron transfer number and the exchange current density of NADH oxidation by SWCNT were characterized by LSV in 1 mM NADH solution in 1× PBS buffer from +0.4 to +1.0 V (vs. reversible hydrogen electrode, RHE) at a scan rate of 10 mV/s with different rotating speed and were calculated based on the Koutecký–Levich equation

$$\frac{1}{i} = \frac{1}{i_K} + \left(\frac{1}{0.620 n_e F A D_{NADH}^{2/3} \upsilon^{-1/6} C}\right)\omega^{-1/2}$$

where i is the measured current density, i_K is the kinetic current density, n_e is the number of electrons transferred, F is the Faraday constant, A is the surface area of the electrode, D_{NADH} is the diffusion coefficient of NADH (D_{NADH} = 2.4 ×10^6 cm^2/s), υ is the kinematic viscosity of the solution (υ = 0.01 cm^2/s for low-concentration aqueous solutions), C is the bulk NADH concentration, and ω is the rotational rate of the electrode.

Cyclic Voltammetry (CV): to identify the existence of NAD$^+$ on SWCNT, the reduction of NAD$^+$ was observed at -1.1 V (vs. Ag/AgCl) in the PBS solution at a scan rate of 100 mV/s. The specific area was evaluated by comparing their capacitance obtained from CV measurements conducted in the PBS buffer from −0.2 to +0.2 V (vs. RHE) at different scan rates (10, 25, 50, 75, 100, 125 mV/s) by using the following equation

$$I = \nu\, C_{dl}$$

where I is the measured current at a set voltage, v is the scan rate, and C_{dl} is the double-layer capacitance, which is directly proportional to the surface area of the electrode exposed to the electrolyte (1).

Electrochemical characterization of enzymatic TMRs. Amperometric measurements: the sensitivity of BHB, glucose, glutamate, G6P, ethanol, *D*-lactate, *L*-lactate, *L*-leucine, cholesterol, glycerol, lactose, and G1P was obtained by amperometric measurements as described previously. The sensor selectivity tests were conducted by stepwise addition of different interference molecules into the PBS, including glucose/BHB, lactate, KCl, NaCl, UA, AA. The target analytes were also introduced into the solutions after the interference. The multiplexed sensor test was conducted by stepwise addition of different targets into the porcine serum.

Characterization of chemical compositions. Scanning transmission electron microscope (S/TEM): the chemical composition of SWCNT after NAD$^+$ (or ATP) integration was characterized

by energy dispersive spectroscopy (EDS) (Oxford X-MaxTEM 100N TLE Windowless SDD 100 mm2) in a Titan S/TEM (FEI). The S/TEM sample was prepared by dissolving NAD^+ (or ATP) integrated SWCNT in ethanol then drop-casting onto a support film grid (Ultrathin Carbon Film on Lacey Carbon Support Film, 400 mesh, Copper, TED PELLA, Inc., CA).

NADH electrochemical reaction model. A two-dimensional microkinetic model in an aqueous solution was constructed and simulated using COMSOL Multiphysics (Ver. 5.5). The model included two layers: the electrode layer and the diffusion layer. A constant potential (E_s0) was applied to the electrode's surface. The reaction of electrochemical oxidation at the electrode's surface proceeded as

$$NADH - 2e^{-} \rightarrow NAD^{+} + H^{+}$$

The kinetic rate of electrochemical reaction was described by the concentration Butler-Volmer equation

$$i = i_0\left(\frac{c_{NADH}}{c_{NADH,0}} exp\left(\frac{\alpha_a F\eta}{RT}\right) - \frac{c_{NAD}}{c_{NAD,0}} exp\left(-\frac{\alpha_c F\eta}{RT}\right)\right)$$

where *i* is the local current density of NADH oxidation at a given location on the porous electrode; i_0 is the exchange current density, determined by analysis of electrochemical data; c_{NADH} and c_{NAD} are local concentrations of NADH and NAD^+; $c_{NADH,0}$ and $c_{NAD,0}$ are the concentrations of NADH and NAD^+ in bulk solution; $\alpha_a = \alpha_c = 0.5$ is the transfer coefficient; *F* is the Faraday constant; *R* is the gas constant; *T* is the temperature; η is over potential, defined as the difference between the applied potential and the standard redox potential of NAD^+/NADH.

Governing differential equations:

$$\frac{\partial c_{NADH}}{\partial t} = D_{NADH}\nabla^2 c_{NADH} = 0$$
$$\frac{\partial c_{NAD}}{\partial t} = D_{NAD}\nabla^2 c_{NAD} = 0$$

At the diffusion layer boundary

$$c_{NADH} = c_{NADH,0}$$
$$c_{NAD} = c_{NAD,0}$$

The length of the diffusion layer (Diff) was defined as the distance between the boundary of the diffusion layer and the boundary of the enzyme layer. A parametric sweep of the diffusion layer length was done to investigate the effect of the diffusion.

Enzymatic electrochemical reaction hybrid model. The model included three layers: the electrode layer, the enzyme layer, and the diffusion layer. The electrode layer was where the electrochemical reaction happened, and it consisted of carbon nanotubes (CNT). The width (CNT_w) of the electrode layer was measured by experiments and the thickness (CNT_h) of the electrode was estimated from the density of the materials used. A layer of enzyme was deposited on the top of the electrode and the enzymatic reaction happened within the enzyme layer. The width of the enzyme layer was considered to be the same as the electrode layer, and the thickness (Elayer_h) was estimated from the density of the materials used. Outside the enzyme layer is the diffusion layer. The length of the diffusion layer (Diff) was defined as the distance between the boundary of the diffusion layer and the boundary of the enzyme layer. The boundary condition at the diffusion layer was considered to be equilibrated with the bulk solution.

A porous electrode module was used to simulate the electrode layer. The specific area (e.g., area_CNT) and the electrode volume fraction (f_s) were obtained by calculations based on the electrode materials' specification sheets. A constant potential (E_s0) was applied to the electrode's surface. The reaction of electrochemical oxidation at the electrode's surface proceeded as

$$NADH - 2e^- \rightarrow NAD^+ + H^+$$

The kinetic rate of electrochemical reaction was described by the concentration Butler-Volmer equation

$$i = i_0\left(\frac{c_{NADH}}{c_{NADH,0}} exp\left(\frac{\alpha_a F\eta}{RT}\right) - \frac{c_{NAD}}{c_{NAD,0}} exp\left(-\frac{\alpha_c F\eta}{RT}\right)\right)$$

where i is the local current density of NADH oxidation at a given location on the porous electrode; i_0 is the exchange current density, determined by analysis of electrochemical data; c_{NADH} and c_{NAD} are local concentrations of NADH and NAD^+; $c_{NADH,0}$ and $c_{NAD,0}$ are the concentrations of NADH and NAD^+ in bulk solution; α_a=α_c=0.5 is the transfer coefficient; F is the Faraday constant; R is the gas constant; T is the temperature; η is over potential, defined as the difference between the applied potential and the standard redox potential of NAD^+/NADH.

A porous matrix module was used to simulate enzymatic layer. The enzymatic reaction proceeded as

$$\beta - hydroxybutyrate + NAD^+ \rightarrow Acetoacetate + NADH$$

The kinetic rate of the enzymatic reaction was described by Michaelis-Menten equation:

$$r_P = -r_S = k_{cat}\frac{c_{E,0} \cdot c_{BHB}}{K_M + c_{BHB}}$$

where r_P is the generation rate of the product; r_S is the consumption rate of the substrate; k_{cat} is the catalytic rate constant of the enzymatic reaction; K_M is the Michaelis constant of the enzyme; $c_{E,0}$ is the initial concentration of enzyme; c_{BHB} is the local concentration of β-hydroxybutyrate.

Governing differential equations:

$$\frac{\partial c_{BHB}}{\partial t} = D_{BHB}\nabla^2 c_{BHB} = 0$$
$$\frac{\partial c_{acac}}{\partial t} = D_{acac}\nabla^2 c_{acac} = 0$$
$$\frac{\partial c_{NADH}}{\partial t} = D_{NADH}\nabla^2 c_{NADH} = 0$$
$$\frac{\partial c_{NAD}}{\partial t} = D_{NAD}\nabla^2 c_{NAD} = 0$$

where c_{BHB}, c_{acac}, c_{NADH}, and c_{NAD} are the local concentrations of β-hydroxybutyrate, acetoacetate, NADH and NAD^+.

At the enzyme layer boundary

$$c_{NADH} = c_{NADH,0}$$

$$c_{NAD} = c_{NAD,0}$$

where $c_{NADH,0}$ and $c_{NAD,0}$ are the concentration of NADH and NAD^+ in bulk solution.

At the diffusion layer boundary

$$c_{BHB} = c_{BHB,0}$$
$$c_{acac} = c_{acac,0}$$

where $c_{BHB,0}$ and $c_{acac,0}$ are the concentration of *β*-hydroxybutyrate and acetoacetate in bulk solution.

A total current of electrochemical reaction was calculated to evaluate the performance of the hybrid system. A parametric sweep of the specific area of porous electrode and the exchange current density was done to investigate the effect of the catalysts' properties. A parametric sweep of the catalytic rate constant (k_{cat}) and the Michaelis constant (K_M) was also done to demonstrate and guide the potential application on different enzymes.

In-vitro biocompatibility test. Cell culture assay: the cell toxicity was evaluated using human foreskin fibroblasts (HFFs). Fabricated devices or components were sterilized (UV for 15 mins) before being incubated in the sterile Dulbecco's Modified Eagle Medium (DMEM) for 7 days at 37 °C to obtain a "conditioned medium". HFFs were cultured in the conditioned medium (10% of fetal bovine serum was added) for 24 hours. The positive control group was created by the addition of AgNO3 into the DMEM to reach a concentration of 10 μg/mL. To quantify the viability, the cells were analyzed by PrestoBlue™ Cell Viability Reagent (Invitrogen) using the fluorescence (plate-reader SynergyMX, Biotech) with excitation wavelength 560 nm and emission wavelength 590 nm. Each study involved two biological replicates and two technical replicates.

Biological sample collection and quantification. Human sample collection: for sweat samples, a subject's inner wrist was first cleaned with deionized water and ethanol, followed by sweat gland stimulation by iontophoresis for 5 min (Macroduct sweat collection system, ELITechGroup Inc.). Then, a sweat collector was attached to the sweating region for 30 min. For whole saliva samples, the collection was performed by using a passive drool method (Saliva Collection Aid, Salimetrics).

Capillary blood measurement: a commercially-available meter (Precision Xtra, Abbott) with BHB and glucose test strips (Abbott) were used to quantify BHB or glucose in capillary blood sampled by fingerprick before saliva and sweat sample collection from human subjects.

Fluorometric/colorimetric assay measurement: BHB and glucose levels from undiluted human sweat and saliva samples, and mice serum samples were quantified with assays (ab180876 and ab65333) performed with a microplate reader in fluorescence intensity detection mode (Omega, BMG LABTECH). Homogenized brain tissue from mice were extracted in a concentration of 1 mg/μL and then deproteinized. *D*-lactate levels from mice brain tissue and serum were quantified with assays (ab83429) performed with a microplate reader in absorbance detection mode (Omega, BMG LABTECH).

Monocolonized mice on fermentable diet. Animals: Mice used for data collection were male germ-free (GF) wild-type Swiss Webster mice, at least 7-8 weeks of age. GF Swiss Webster mice were purchased from Taconic Farms and bred in flexible film isolators at the UCLA Goodman-Luskin Microbiome Center Gnotobiotics Core Facility. All experimental procedures were carried out in accordance with US NIH guidelines for the care and use of laboratory animals and approved by the University of California, Los Angeles Animal Research Committee, under the ARC protocol number 2015-079.

Treatment: GF mice were exited from flexible film isolators into autoclaved cages with sterile water containing 100 μg/mL gentamicin and habituated to the new environment for one week. Mice were then single housed for the duration of the experiment to measure individual diet intake. Mice (N = 5) were mono-colonized by one 200 μL oral gavage of turbid bacterial culture of B. thetaiotaomicron VPI-5482 (~109 CFU/mL). Control mice (N = 3) had one 200 μL oral gavage

of growth media and remained GF. All mice were fed a carbohydrate-restricted diet that contains levan, a host non-digestible polysaccharide, as its only carbohydrate source (63% fat, 17% protein, 10% levan; Inotiv-Teklad) for 8 days. After an overnight fast, they were exposed to levan diet for 2 hours before taking the diet away. After 2 hours, they were sacrificed, and serum and brain tissue were collected.

Dextran sulfate sodium (DSS) and fiber mix treatment in mice. Animals: 8-9 week old wild-type C57BL/6J mice from Jackson Laboratory were used. Mice were housed on a 12-h light-dark schedule in a temperature-controlled (22-25˚C) and humidity-controlled environment with ad libitum access to water and sterile "breeder" chow (Lab Diets 5K52) or experimental diets as described below. All experimental procedures were carried out in accordance with US NIH guidelines for the care and use of laboratory animals and approved by the University of California, Los Angeles Animal Research Committee, under the ARC protocol number 2015-079.

Treatment: WT C57BL/6J mice (*N* = 8-10) were treated with 3% DSS (MP Biomedicals) dissolved in sterile water ad libitum for 7 days. On day 7, mice were fasted overnight and given 200 uL of fiber mix twice by oral gavage 12 and 6 hours before they were sacrificed. During this time, mice did not have access to normal chow. The fiber mix (20 mg/mL) consisted of fructooligosaccharides (Sigma-Aldrich), inulin from chicory (Sigma-Aldrich), crystalline cellulose (Sigma-Aldrich), gum arabic from Acacia Tree (Sigma-Aldrich), wheat, pea, potato, and apple fiber from J. Rettenmaier USA LP, orange (citrus) fiber from Citri-Fi Naturals, oat (NuNaturals), acacia (Nutricost organic), and psyllium husk (It's just).

In-vivo TMR array characterization in mice. Animals: mixed gender, body weight 20-28 g, 3-to-4-month-old mice were used in this study. All animal studies were performed according to the protocols approved by the University of California, Los Angeles Animal Research Committee, under the ARC protocol number 2019-019. The experimental sample size (8 mice in total) was determined by a similar study. The methods were carried out in accordance with the relevant guidelines and regulations in full compliance with the ARRIVE (Animal Research: Reporting of in vivo Experiments) guidelines 2.0.

In-vivo test in mice: before each study, animals were weighed to make sure that their body weights were over 20 g. Mice were anesthetized by isoflurane via inhalation (Isoflurane vaporizer, Somni Inc.). The induction of the anesthesia was done with 3-5% isoflurane no longer than 2 min and the maintenance of the anesthesia was done with 0.5-2% isoflurane. Toe pinch was used to validate the state of anesthesia every 15 min throughout the entire procedure. A pneumotach (biopac) was connected to the mouthpiece of the anesthetic system to monitor the airway pressure change. Once the animal was fully anesthetized, one incision was made to the skin of the base of tail for subcutaneous placement of the recording sensors. One sensor array with two types of sensors (BHB and glucose), one reference electrode and one control electrode will be placed topically near the abductor caudae dorsalis muscle close to the base of the tail. The skin will cover and stabilize the sensor arrays and biocompatible Tegaderm tape (3M) was used to further stabilize the sensor at the recording sites. After being stabilized, the sensor arrays were connected to the potentiostat, and the signals were collected at a sampling rate of 10 Hz.

The recording session had two phases: the pre-nutrient delivery phase and the nutrient delivery phase. After the sensor placement, the sensors were stabilized subcutaneously to reach the equilibrium status in vivo and establish the pre-nutrient delivery baseline. After the pre-nutrient delivery baseline was established, one dose of BHB (2.5 g/kg in saline solution) or one dose of glucose (1.0 g/kg in saline solution) was given to the animal via tail-vein injections. After around 20 min of recording, another dose of BHB (2.5 g/kg in saline solution) was given to the animal in a similar way. The recording was stopped 20 min after the second dose.

In-vivo TMR array characterization in rat. Animals: mixed gender, body weight 250-350 g rats were used in this study. All animal studies were performed according to the protocols approved by the University of California, Los Angeles Animal Research Committee, under the ARC protocol number 2021-011. The methods were carried out in accordance with the relevant guidelines and regulations in full compliance with the ARRIVE (Animal Research: Reporting of in vivo Experiments) guidelines 2.0.

In-vivo test in rat: before each study, animals were weighed to make sure that their body weights were over 250 g. Rat were anesthetized by isoflurane via inhalation (Isoflurane vaporizer, Somni Inc.). The induction of the anesthesia was done with 3-5% isoflurane no longer than 2 min and the maintenance of the anesthesia was done with 0.5-2% isoflurane. Toe pinch was used to validate the state of anesthesia every 15 min throughout the entire procedure. Once the animal was fully anesthetized, a tail vein catheter was used for injection and one incision was made to the skin of the base of tail for subcutaneous placement of the recording sensors. One sensor array with two types of sensors (*D*-lactate and *L*-lactate) and one reference electrode will be placed topically near the abductor caudae dorsalis muscle close to the base of the tail. The skin will cover and stabilize the sensor arrays and biocompatible Tegaderm tape (3M) was used to further stabilize the sensor at the recording sites. After being stabilized, the sensor arrays were connected to the potentiostat, and the signals were collected at a sampling rate of 10 Hz.

For brain monitoring, the anesthesia procedure follows the same and a tail vein catheter was used. The rat's head was secured in a stereotaxic frame. A midline scalp incision was made to expose the skull, and a craniotomy was performed using a high-speed dental drill to remove a portion of the skull, thereby exposing the underlying brain tissue. Care was taken to minimize damage to the brain surface and avoid excessive bleeding. After the skull was removed, the dura mater was carefully excised with fine forceps to fully expose the cortical surface. The exposed brain was continuously irrigated with sterile saline to prevent desiccation and maintain tissue integrity. *D*-lactate sensor, reference electrode, and counter electrode will be placed topically on the top of the cortical surface. After being stabilized, the sensor arrays were connected to the potentiostat, and the signals were collected at a sampling rate of 10 Hz.

The recording session had two phases: the pre-metabolite delivery phase and the metabolite delivery phase. After the sensor placement, the sensors were stabilized subcutaneously to reach the equilibrium status in vivo and establish the pre-metabolite delivery baseline. After the pre-nutrient delivery baseline was established, one dose of *D*-lactate (1.0 g/kg in saline solution) was given to the animal via tail-vein catheter. The recording was stopped 20 min after the dose.

Surveys for metabolic pathway. The metabolic pathway surveys were summarized from Roche Biochemical Pathways (4th Edition, Part 1) and (2). All metabolites were screened for linkability to a cofactor-involved oxidoreductase reaction through an optional single intermediation step.

Surveys for oxidoreductase library and measurable biomarkers. Enzyme data source: the BRENDA (The Comprehensive Enzyme Information System) database (3) served as the resource for estimating the total number of oxidoreductases. Focusing on enzymes categorized under the EC number 1, specifically those identified as oxidoreductases, the search criteria included enzymes with names containing peroxidase, reductase, dehydrogenase, oxidase, oxygenase, and hydroxylase.

Categorization of enzymatic reactions and measurable biomarkers: enzymatic reactions producing hydrogen peroxide, H_2O_2, from the electron donor oxygen O_2 were labeled as non-cofactor-based catalytic reactions, while the remaining reactions involving NAD-based cofactors or other cofactors were considered cofactor-based catalytic reactions. The analyte of enzymatic reactions that involve NAD-based cofactors or oxygen, O_2 (without NAD-based cofactors' participation), was counted as a measurable biomarker.

Wireless printed circuit board (PCB) module. The PCB module was adapted from our previous design (4), with the LMP91000 chip now programmed to apply 0 V across the working and reference (Ag/AgCl) electrodes connected to the sensor.

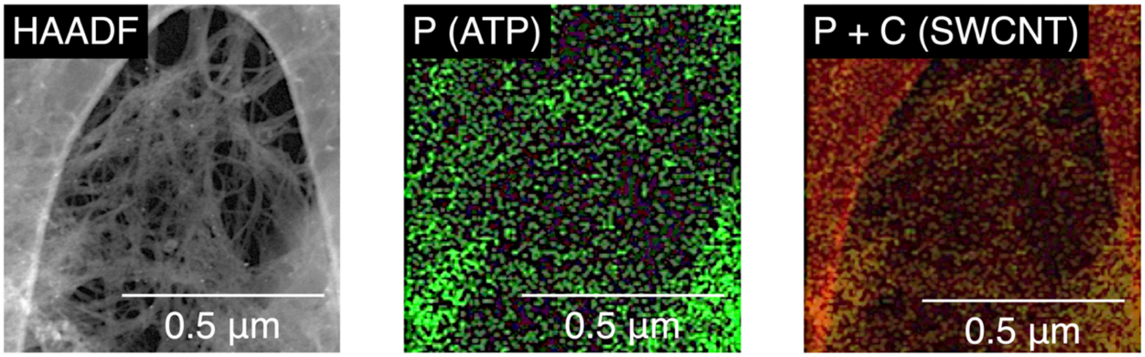

Fig. S1. S/TEM-EDS images of ATP integrated TMR. HAADF, high-angle annular dark-field imaging. P, phosphorus, the signature element for ATP. C, carbon.

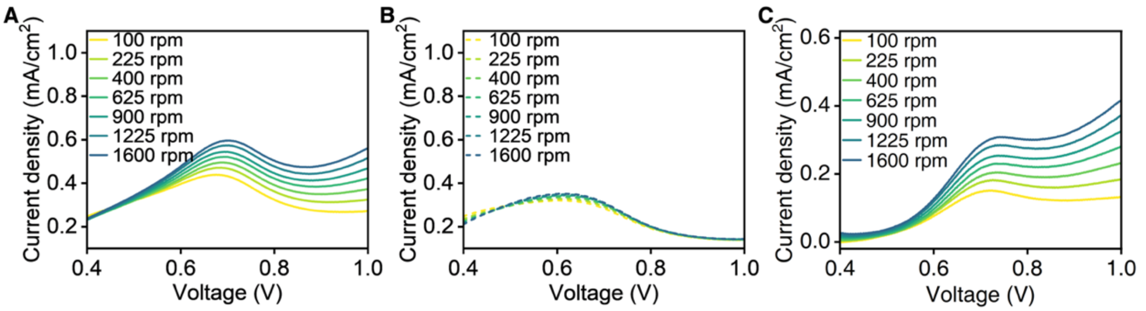

Fig. S2. Rotating disk electrode measurement of NADH oxidation. Linear scanning voltammograms (vs. RHE) of the SWCNT-based NADH sensor at (A) 1 mM NADH supplemented 1 × PBS, (B) 1 × PBS (baseline measurement), and (C) baseline-subtracted 1 mM NADH response at various rotational speeds.

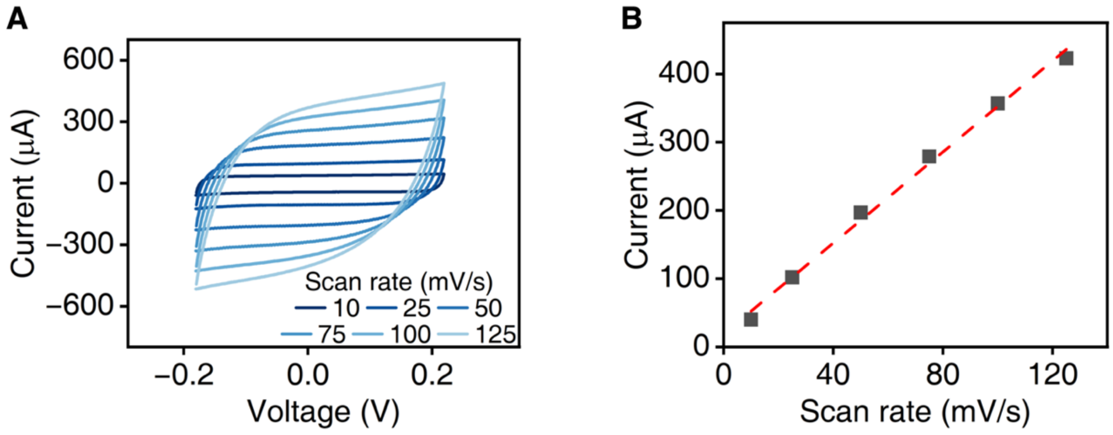

Fig. S3. Specific capacitance of SWCNT-based NADH sensor. (A) Cyclic voltammetry of the SWCNT-based NADH sensor at varying scanning rates. (B) Corresponding plot of current vs. scan rate at 0.1 V (vs. RHE).

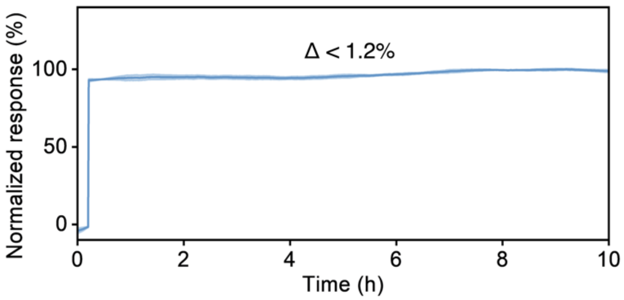

Fig. S4. Real-time NADH measurements. A 10-h long real-time amperometric measurement performed with TMRs in PBS supplemented with a 150 μM NADH increase from baseline (0 μM). Normalized response = $(I - I_{Baseline})/(I_{Max} - I_{Baseline})$, error band indicates standard deviations (N = 3).

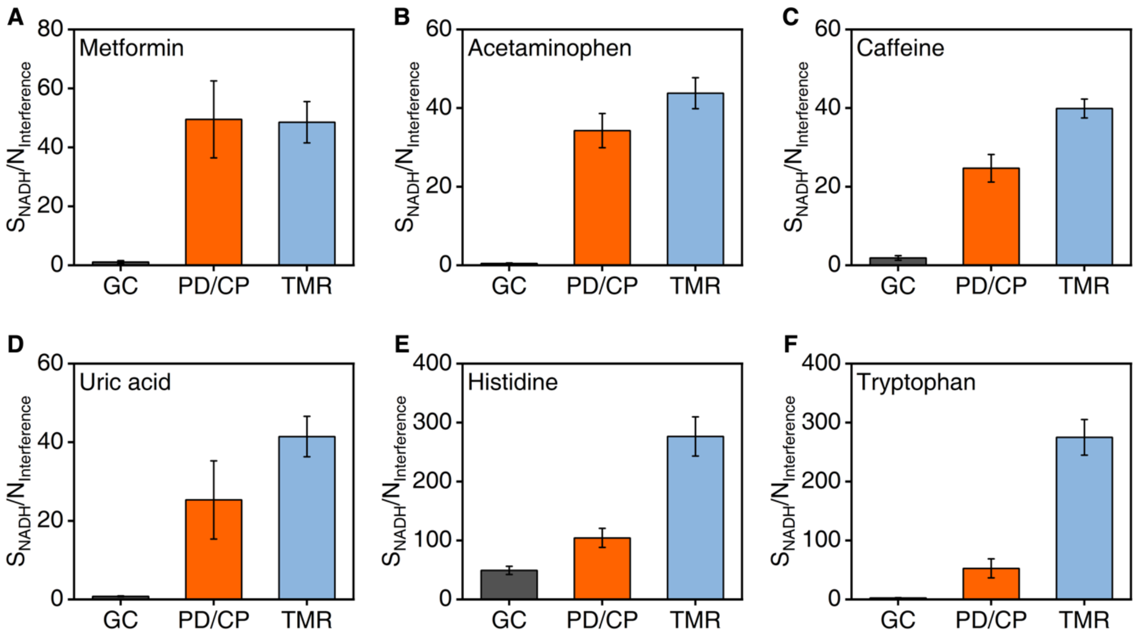

Fig. S5. SNR comparison for various electroactive metabolites. (A-F) SNR comparison among GC, PD/CP, and TMR for metformin (A), acetaminophen (B), caffeine (C), uric acid (D), histidine (E), and tryptophan (F). *N* = 3 for each tested sensor. Error bars indicate standard deviations.

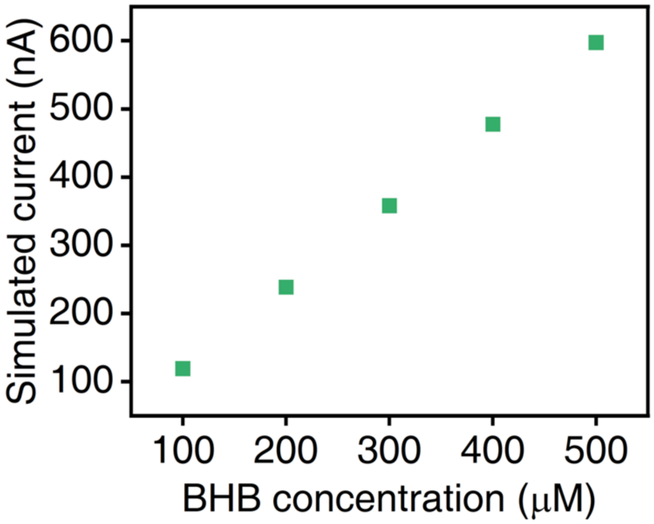

Fig. S6. BHB response simulation. Simulated calibration curve from the enzymatic electrochemical reaction hybrid model, showing a sensitivity difference within an order of magnitude compared to the experimental BHB calibration curve in Fig. 3B, demonstrating reasonable agreement between simulation and experiment.

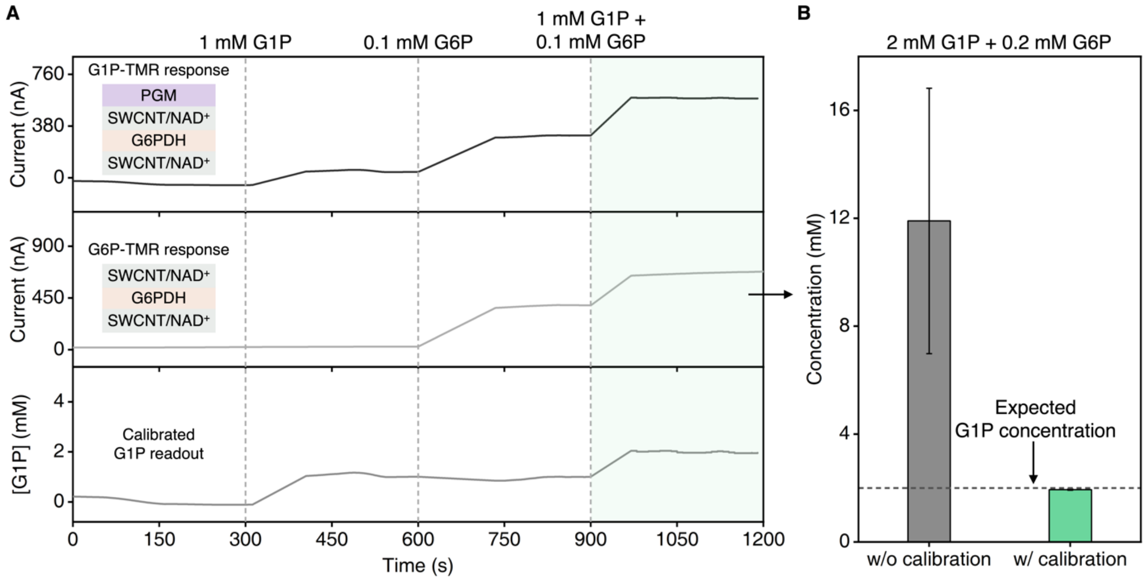

Fig. S7. Upstream metabolite calibration test. (A) Representative real-time amperometric measurements (top two panels) and calibrated G1P readout (bottom panel) with a G1P-TMR (primary TMR) and a G6P-TMR (secondary TMR) simultaneously in the same PBS buffer environment. Dotted lines indicate sample spiking timepoints. (B) Estimation of the end-point G1P (upstream metabolite) concentration level by G1P sensors (*N* = 3) with and without concentration calibration by G6P sensors (*N* = 3) for the buffer solution containing 2 mM G1P and 0.2 mM G6P. Error bars indicate standard deviations.

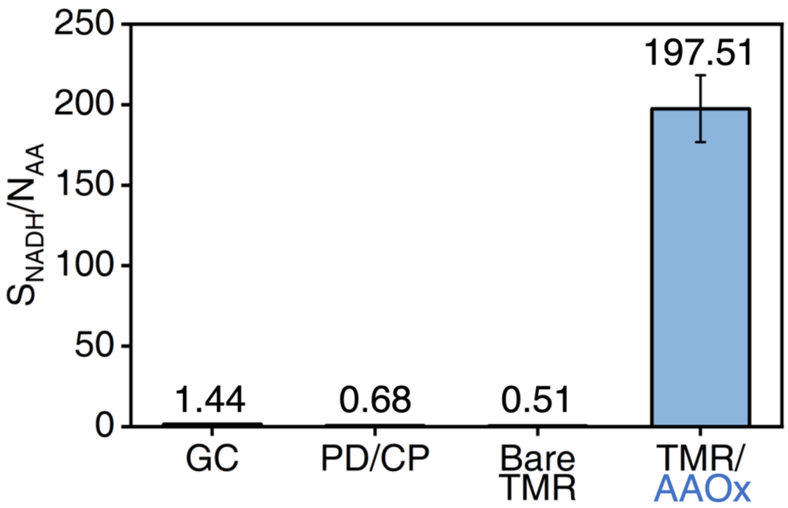

Fig. S8. SNR comparison for AA. SNR comparison among GC, PD/CP, bare TMR, and AAOx-coupled-TMR for AA. *N* = 3 for each tested sensor. Error bars indicate standard deviations.

A

Number	1	2	3	4	5	6
Interference	BHB	*L*-Lactate	KCl	NaCl	Uric acid	AA
Conc. (µM)	50	5000	2400	10000	59	100

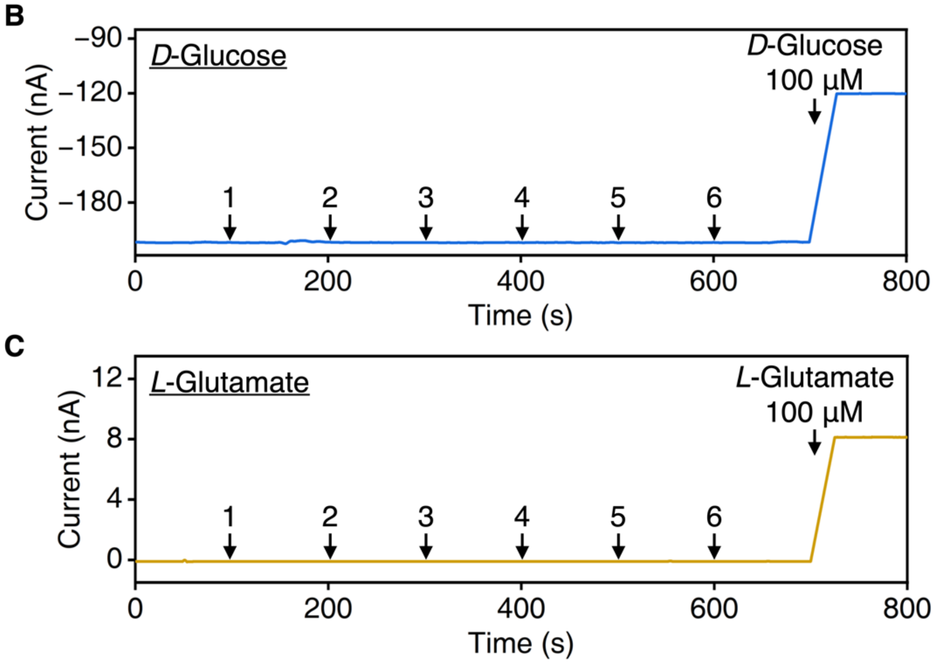

Fig. S9. Selectivity test with various metabolites and ions. (A) Table of common interferences in biofluids (e.g., sweat and saliva). (B) Real-time amperometric selectivity study with a representative *D*-glucose-TMR. (C) Real-time amperometric selectivity study with a representative *L*-glutamate-TMR. The introduction timepoints for the interferences and target analytes are indicated by the arrows.

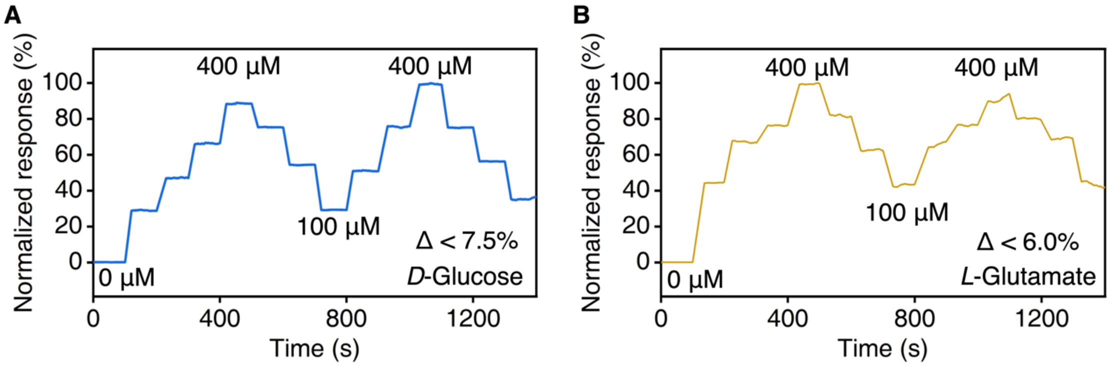

Fig. S10. Reversibility tests. Real-time amperometric measurement with various concentrations of the analyte in 1× PBS suggested that the high level of reversibility of (A) *D*-glucose-TMR and (B) *L*-glutamate-TMR.

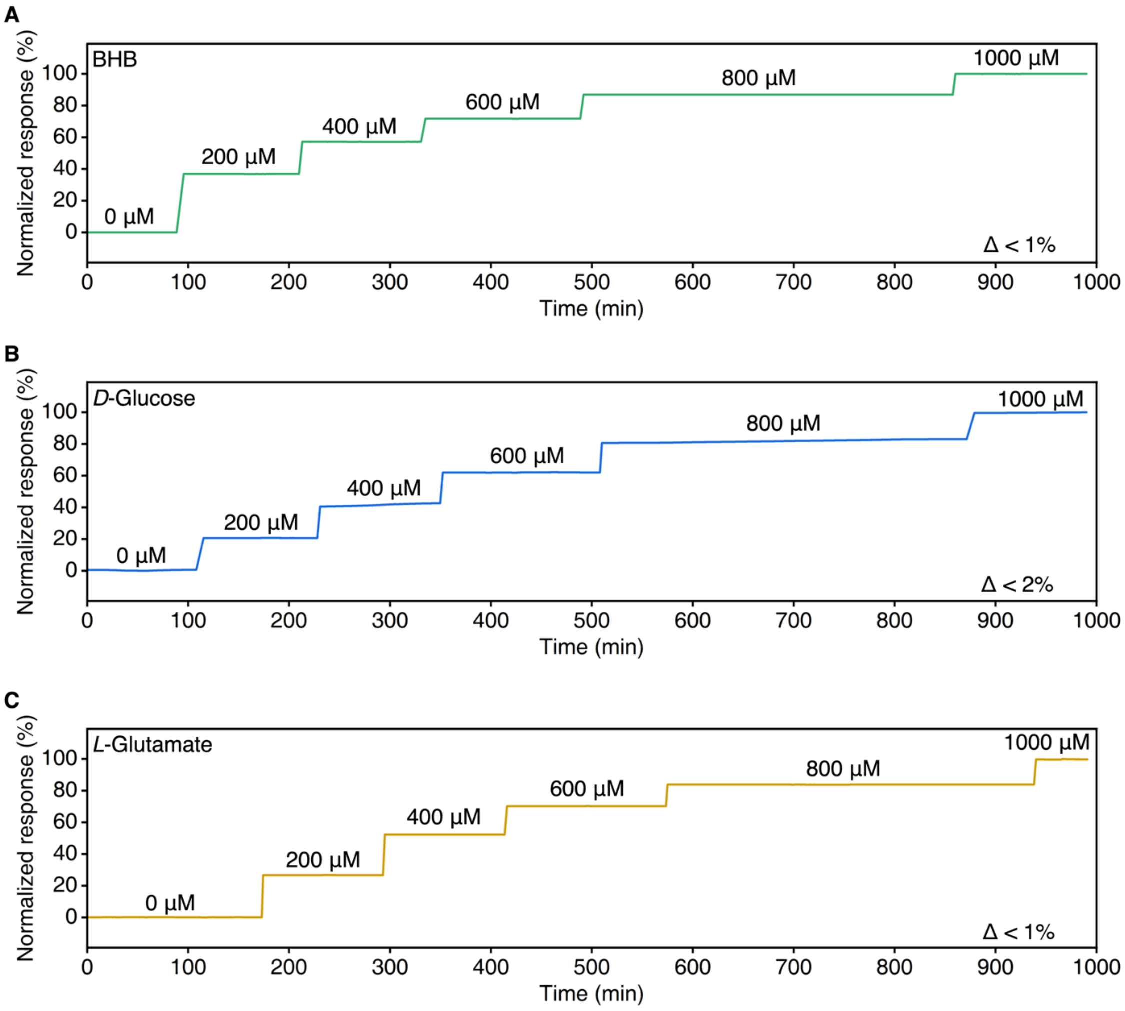

Fig. S11. Real-time amperometric measurement with fouling agent. A 1000-minute real-time amperometric measurement performed with (A) BHB-TMR, (B) *D*-glucose-TMR, and (C) *L*-glutamate-TMR in 1× PBS buffer with 20 mg/mL BSA. The analyte concentrations are annotated for the corresponding time windows.

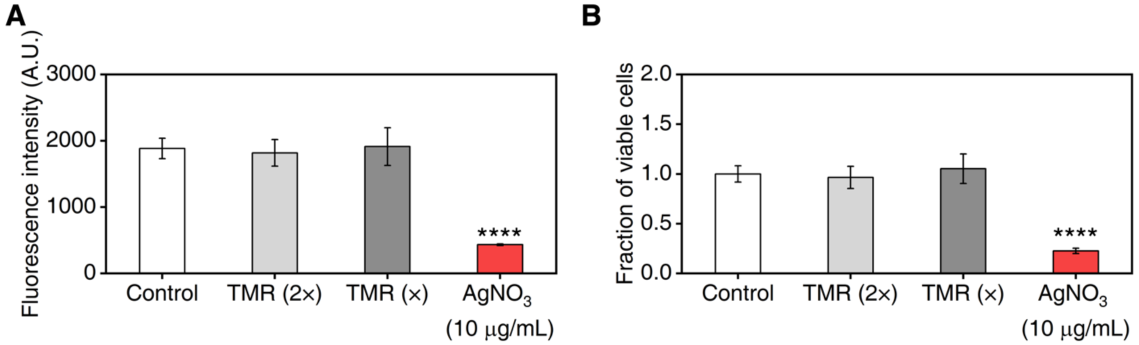

Fig. S12. Ex-vivo biocompatibility tests. The results of cellular viability test in terms of (A) fluorescence intensity and (B) fraction of viable cells. 4 TMRs were immersed into 2 mL (2×) or 4 mL (×) of the medium. The cell toxicity was evaluated based on the human foreskin fibroblasts (HFF) system. Error bars indicate standard deviations. Statistical significance and *P* values were determined by one-way ANOVA. **** $P < 0.0001$.

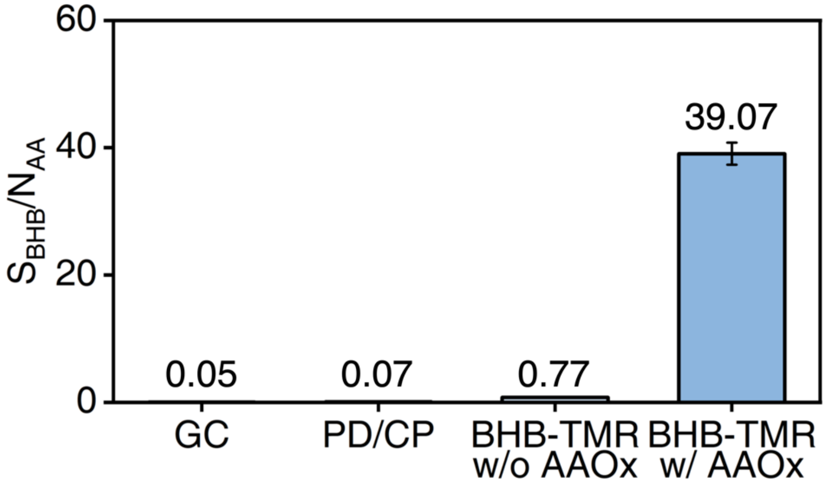

Fig. S13. SNR comparison among GC, PD/CP, bare TMR, and AAOx-coupled-TMR for AA in saliva. BHB-DH solution was drop-cast onto GC and PD/CP electrodes to fabricate BHB sensors. GC and PD/CP were tested in saliva with 1 mM NAD^+ to ensure sufficient NAD^+ supply. *N* = 3 for each tested sensor. Error bars indicate standard deviations.

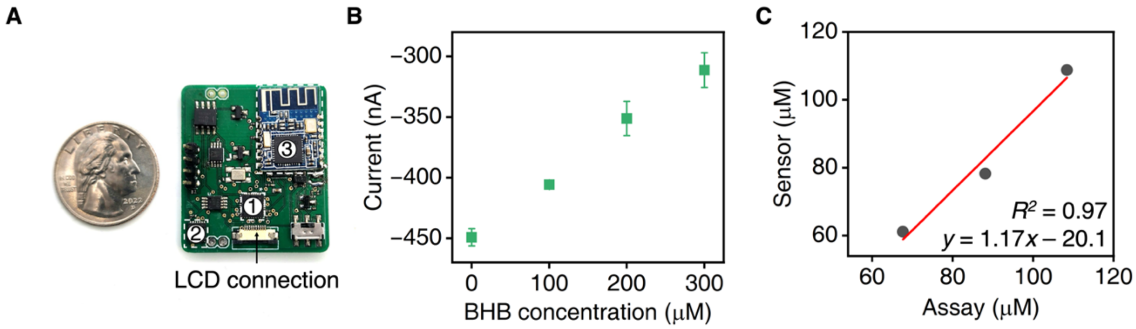

Fig. S14. BHB determination in sweat using wireless sensing system. (A) Photograph of the custom-developed wireless readout board next to a U.S. quarter. The components are (1) microcontroller unit, MCU, (2) potentiostat chip, and (3) Bluetooth chip. (B) BHB-TMR (N = 3) calibration response measured by the custom-developed readout board in 1× PBS buffer. Error bars indicate standard deviations. (C) The determination of BHB concentration in sweat by TMR-based wireless sensing system vs. the corresponding standard assay-quantified BHB concentrations.

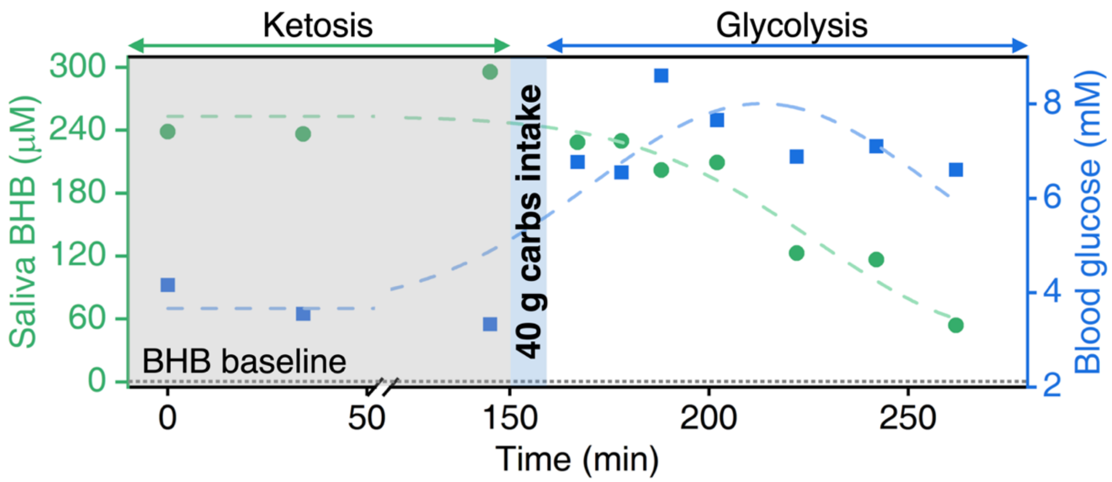

Fig. S15. Healthy subject fasting study. Saliva BHB monitoring with BHB-TMR and blood glucose monitoring with commercialized glucose meter for a two-day fasting healthy subject before and after consuming a high carbohydrate beverage. The baseline salivary BHB levels were measured using a standard assay prior to beginning the fasting period.

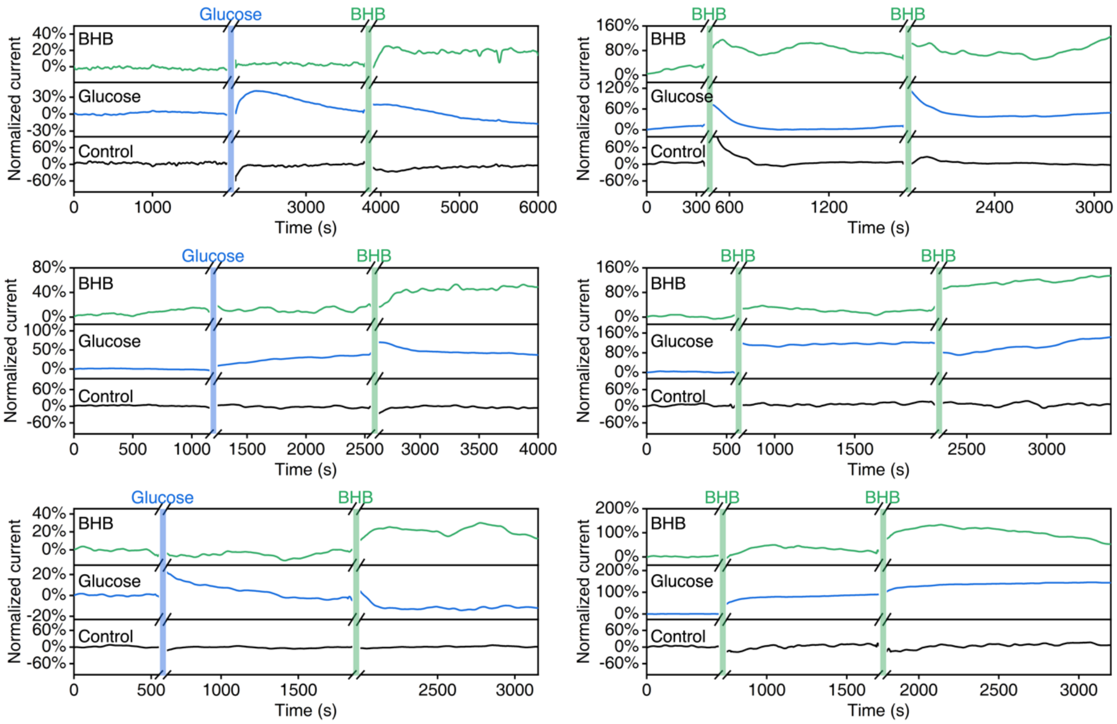

Fig. S16. In-vivo multiplexed TMRs monitoring. Left: Three multiplexed continuous monitoring experiments with one injection of glucose and one injection of BHB. Right: Three multiplexed continuous monitoring experiments with dual injections of BHB.

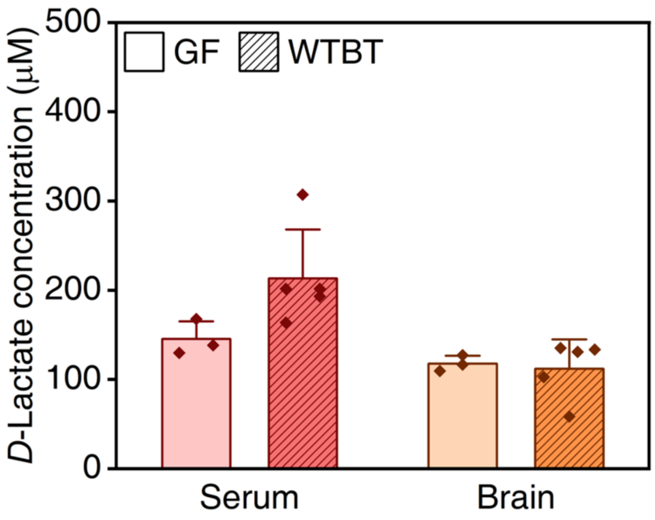

Fig. S17. Effects of bacterial fermentation on *D*-lactate levels. *D*-lactate concentrations in serum and brain tissue extract of germ-free mice (GF) vs. *B. thetaiotaomicron* monocolonized mice (WTBT). Statistical significance and *P* values were determined by two-tailed unpaired Student's t test. Serum: *P* = 0.0895. Brain: *P* = 0.788.

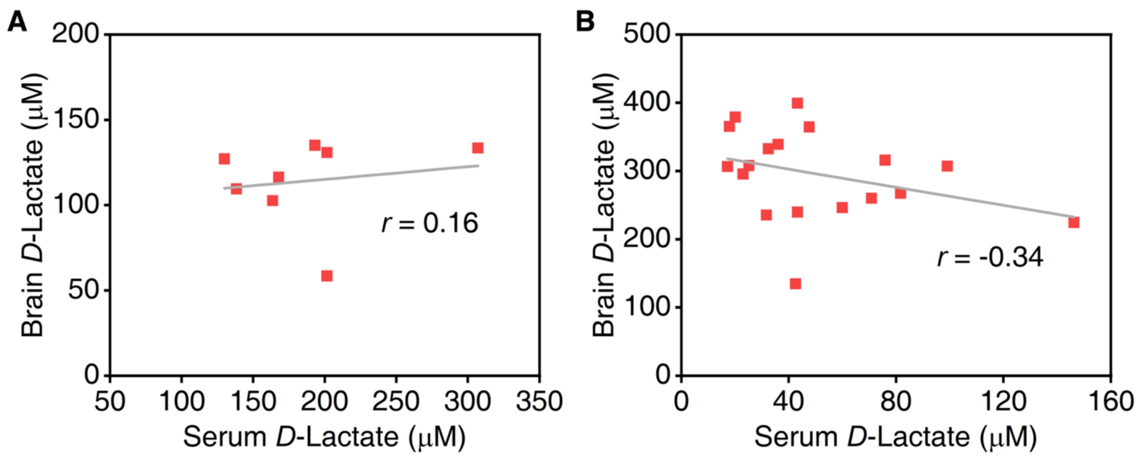

Fig. S18. *D*-lactate concentration correlations in serum and brain. Serum-brain tissue extract *D*-lactate concentration correlations for mice in the GF vs. WTBT study (A) and DSS treatment study (B).

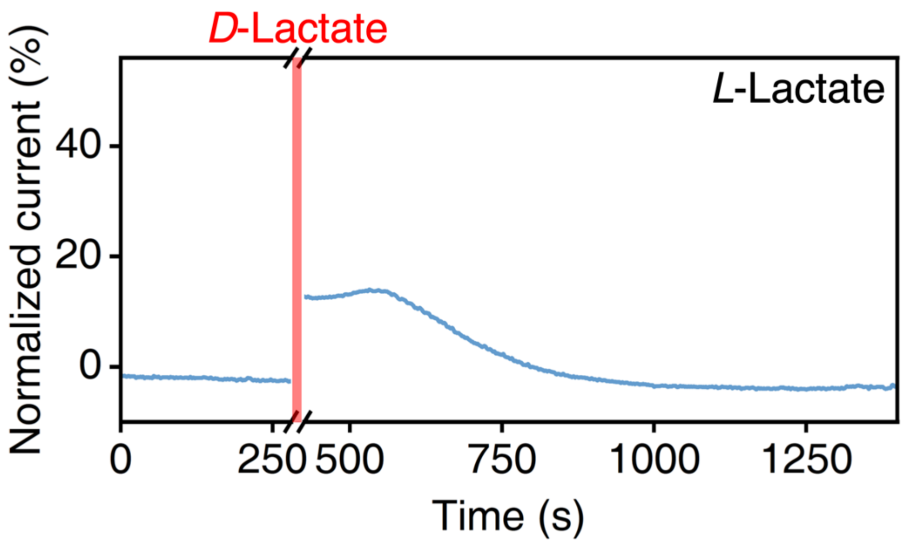

Fig. S19. In-vivo multiplexed TMR monitoring. Continuous monitoring of *L*-lactate and *D*-lactate (shown in Fig. 4H) with one injection of *D*-lactate.

Table S1. Physiological ranges of various analytes in blood and saliva (physiological origin exceptions marked in parenthesis).

Analyte	Blood concentration	Saliva concentration	Reference
BHB	0.1 – 6.3 mM	0 – 360 μM	This paper
D-Glucose	4 – 17 mM	0.03 – 1 mM	(5)
L-Glutamate	60 – 80 μM	18 – 49 μM	(6, 7)
G6P	0.1 – 34.58 mM (intracellular)	Not reported	(8, 9, 10)
Ethanol	10 – 70 mM	10 – 60 μM	(11)
D-Lactate	30 – 180 μM	Not reported	(12)
L-Lactate	1.21 – 1.47 mM	90 –130 μM	(13)
L-Leucine	150 – 500 μM	10 – 300 μM	(14, 15)
Cholesterol	4.8 – 5.4 μM	0.02 – 5.46 μM	(16)
Glycerol	55 – 228 μM	Not reported	(17)
Lactose	10 – 60 μM	Not reported	(18)
G1P	0.1 – 53.82 mM (intracellular)	0.63 – 2.01 μM	(9, 10, 19)

Dataset S1 (separate file). Survey of measurable metabolites in metabolic pathways. A survey of the metabolites detectable by enzymatic reactions involving NAD-based cofactors is summarized in the separated Excel file.

Dataset S2 (separate file). Survey of measurable metabolites by single-step oxidoreductase catalysis. A survey of the metabolites detectable by enzymatic reactions involving O_2, NAD-based cofactors, or both is summarized in the separated Excel file.

SI References

1. C. C. McCrory, S. Jung, J. C. Peters, T. F. Jaramillo, Benchmarking heterogeneous electrocatalysts for the oxygen evolution reaction. *JACS*, 135(45), 16977-16987 (2013).
2. Schomburg, G. Michal, *Biochemical pathways : an atlas of biochemistry and molecular biology.* (John Wiley & Sons, Hoboken, N.J., ed. 2nd, 2012), pp. xi, 398 p.
3. A. Chang, L. Jeske, S. Ulbrich, J. Hofmann, J. Koblitz, I. Schomburg, M. Neumann-Schaal, D. Jahn, D. Schomburg, BRENDA, the ELIXIR core data resource in 2021: new developments and updates. *Nucleic Acids Res.* 49, D498–D508 (2021).
4. X. Cheng, B. Wang, Y. Zhao, H. Hojaiji, S. Lin, R. Shih, H. Lin, S. Tamayosa, B. Ham, P. Stout, K. Salahi, Z. Wang, C. Zhao, J. Tan, S. Emaminejad, A mediator-free electroenzymatic sensing methodology to mitigate ionic and electroactive interferents' effects for reliable wearable metabolite and nutrient monitoring. *Adv. Funct. Mater.* 30, 1908507 (2020).
5. P. Abikshyeet, V. Ramesh, N. Oza, Glucose estimation in the salivary secretion of diabetes mellitus patients. *Diabetes Metab. Syndr. Obes. Targets Ther.* 5, 149-154 (2012).
6. W. Bai, W.-L. Zhu, Y.-L. Ning, P. Li, Y. Zhao, N. Yang, X. Chen, Y.-L. Jiang, W.-Q. Yang, D.-P. Jiang, L.-Y. Chen, Y.-G. Zhou, Dramatic increases in blood glutamate concentrations are closely related to traumatic brain injury-induced acute lung injury. *Sci. Rep.* 7, 5380 (2017).
7. A. Scinska-Bienkowska, E. Wrobel, D. Turzynska, A. Bidzinski, E. Jezewska, H. Sienkiewicz-Jarosz, K. Golembiowska, W. Kostowski, A. Kukwa, A. Plaznik, P. Bienkowski, Glutamate concentration in whole saliva and taste responses to monosodium glutamate in humans. *Nutr. Neurosci.* 9, 25–31 (2006).
8. A. Zhu, R. Romero, H. R. Petty, An enzymatic fluorimetric assay for glucose-6-phosphate: Application in an in vitro Warburg-like effect. *Anal. Biochem.* 388, 97–101 (2009).
9. E. Guedon, M. Desvaux, H. Petitdemange, Kinetic Analysis of Clostridium cellulolyticum Carbohydrate Metabolism: Importance of Glucose 1-Phosphate and Glucose 6-Phosphate Branch Points for Distribution of Carbon Fluxes Inside and Outside Cells as Revealed by Steady-State Continuous Culture. *J. Bacteriol.* 182, 2010–2017 (2000).
10. D. P. Aiello, L. Fu, A. Miseta, D. M. Bedwell, Intracellular Glucose 1-Phosphate and Glucose 6-Phosphate Levels Modulate Ca2+ Homeostasis in Saccharomyces cerevisiae. *J. Biol. Chem.* 277, 45751–45758 (2002).
11. K. E. McColl, B. Whiting, M. R. Moore, A. Goldberg, Correlation of ethanol concentrations in blood and saliva. *Clin. Sci.* 56, 283–286 (1979).
12. C. W. Ludvigsen, J. R. Thurn, G. L. Pierpont, J. H. Eckfeldt, Kinetic enzymic assay for D(-)-lactate, with use of a centrifugal analyzer. *Clin. Chem.* 29, 1823–1825 (1983).
13. É. Tékus, M. Kaj, E. Szabó, N. Szénási, I. Kerepesi, M. Figler, R. Gábriel, M. Wilhelm, Comparison of blood and saliva lactate level after maximum intensity exercise. *Acta Biol. Hung.* 63, 89–98 (2012).
14. N. Yoshii, K. Sato, R. Ogasawara, Y. Nishimura, Y. Shinohara, S. Fujita, Effect of Mixed Meal and Leucine Intake on Plasma Amino Acid Concentrations in Young Men. *Nutrients* 10, 1543 (2018).
15. A. M. Casas-Ferreira, M. D. Nogal-Sánchez, E. Rodríguez-Gonzalo, B. Moreno-Cordero, J. L. Pérez-Pavón, Determination of leucine and isoleucine/allo-isoleucine by electrospray ionization-tandem mass spectrometry and partial least square regression: Application to saliva samples. *Talanta* 216, 120811 (2020).
16. S. Karjalainen, L. Sewón, E. Soderling, B. Larsson, I. Johansson, O. Simell, H. Lapinleimu, R. Seppänen, Salivary Cholesterol of Healthy Adults in Relation to Serum Cholesterol Concentration and Oral Health. *J. Dent. Res.* 76, 1637–1643 (1997).
17. J. Lebeck, B. Brock, Plasma glycerol levels in men with hypertriglyceridemia. *Scand. J. Clin. Lab. Invest.* 81, 298–302 (2021).

18. G. Pimentel, K. J. Burton, M. Rosikiewicz, C. Freiburghaus, U. Von Ah, L. H. Münger, F. P. Pralong, N. Vionnet, G. Greub, R. Badertscher, G. Vergères, Blood lactose after dairy product intake in healthy men. *Br. J. Nutr.* 118, 1070–1077 (2017).
19. M. Tsuruoka, J. Hara, A. Hirayama, M. Sugimoto, T. Soga, W. R. Shankle, M. Tomita, Capillary electrophoresis-mass spectrometry-based metabolome analysis of serum and saliva from neurodegenerative dementia patients. *Electrophoresis* 34, 2865–2872 (2013).
